# Gene-gene and gene-environment interaction data for platinum-based chemotherapy in non-small cell lung cancer

**DOI:** 10.1038/sdata.2018.284

**Published:** 2018-12-11

**Authors:** Lei-Yun Wang, Jia-Jia Cui, Jun-Yan Liu, Ao-Xiang Guo, Zhen-Yu Zhao, Ying-Zi Liu, Ji-Chu Wu, Min Li, Cheng-Ping Hu, Yang Gao, Hong-Hao Zhou, Ji-Ye Yin

**Affiliations:** 1 Department of Clinical Pharmacology, Xiangya Hospital, Central South University, Changsha 410008, P. R. China; 2 Institute of Clinical Pharmacology, Central South University; Hunan Key Laboratory of Pharmacogenetics, Changsha 410078, P. R. China; 3 Hunan Provincial Gynecological Cancer Diagnosis and Treatment Engineering Research Center, Changsha 410078, P. R. China; 4 Department of orthopaedics, The First Affiliated Hospital of the University of South China, Hengyang 421001, P. R. China; 5 Department of Respiratory Medicine, Xiangya Hospital, Central South University, Changsha 410008, P. R. China; 6 Department of Thoracic Surgery, Xiangya Hospital, Central South University, Changsha 410008, Changsha, P.R. China; 7 Department of Cardiovascular, Central Hospital of Shaoyang, Shaoyang 422000, P. R. China

**Keywords:** Genotype, Cancer genetics

## Abstract

Gene-gene (GXG) and gene-environment (GXE) interactions play important roles in pharmacogenetics study. Simultaneously incorporating multiple single nucleotide polymorphisms (SNPs) and clinical factors is needed to explore the association of their interactions with drug response and toxicity phenotypes. We genotyped 504 SNPs in a total of 490 Chinese non-small cell lung cancer (NSCLC) patients, and the correlation of GXG and GXE interactions with platinum-based chemotherapeutic efficacy and safety were analyzed. In this data descriptor, we shared our data set which could help others to reuse them. All kinds of file types needed for GXG and GXE analysis were supplied. The process of genotyping and data analysis was also introduced step by step.

## Background and summary

Univariate analysis strategy examining the association of genetic polymorphisms with complex quantitative traits is widely used^1–3^. However, a number of studies show that single variation is far from satisfactory to explain complex phenotypic characteristics^4–6^. For pharmacogenomics investigation, analyzing a single polymorphism at a time is also not sufficient to explain drug efficacy and safety^4,7^. To explore the multivariate contribution to drug response and toxicity phenotypes, we conducted an association study by incorporating multiple single nucleotide polymorphisms (SNPs) and clinical factors simultaneously.^4^ Platinum-based chemotherapeutic response and toxicity in the non-small cell lung cancer (NSCLC) was selected as our investigated phenotypes. Lung cancer ranks one of the tumors with high mortality rate in the world, it consists of NSCLC and small cell lung cancer (SCLC) in histology according to World Health Organization (WHO) classification.^8,9^ Although immune and molecular target therapy is developing rapidly, chemotherapy is still a major treatment choice for NSCLC patients with platinum-based doublet as the first-line regimen^10,11^. One of the challenges for successful treatment is the remarkable inter-individual difference of drug response and toxicity ^12–14^. Only a part of patients can benefit from platinum-based chemotherapy, unfortunately, the contribution of genetic and environment factors to them still remains largely unknown^15,16^. We proposed that the combination of multiple genetic and environmental factors could contribute to the inter-individual variation.

We genotyped 504 SNPs in a total of 490 Chinese NSCLC patients.^4^ Based on these SNPs, we explored the effects of gene-gene (GXG) and gene-environment (GXE) interactions on platinum-based chemotherapeutic response and toxicity. Our results showed that some SNPs previously identified as “negative” were in fact significantly related to the phenotype when conducting interaction analysis. In addition, environment also plays an important role in the drug response through GXE interactions. Some significant results were validated in another cohort. The main purpose of this data descriptor is to share our data set. All kinds of file types needed for GXG and GXE analysis were supplied. Moreover, we provided an analytical method to analyze the correlation of GXG and GXE interaction to complex phenotype in pharmacogenomic research.

## Methods

### Study design

**Figure 1:**
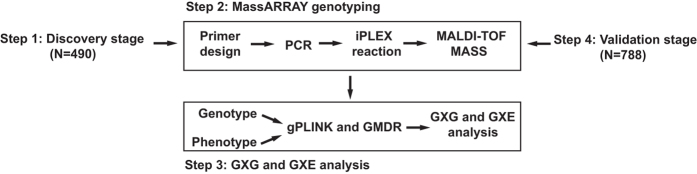
Flow diagram of study design.

As our previous study introduced, the discovery stage enrolled 490 platinum-based treatment NSCLC patients^4^. To enrolled genes which may contribute to platinum-based chemotherapy, 504 SNPs located in 185 genes which are mainly involved in DNA repair, drug transport, apoptosis and detoxification pathways were genotyped.^4^ Then, the correlations of GXG and GXE interaction with drug response were analyzed. 9 pairs of SNP-SNP interactions and 15 groups of multiple gene-gene and gene-environment interactions were found significantly associated with platinum-based chemotherapy in the discovery stage. 16 SNPs were included among these interactions. They were further genotyped in the validation stage including another 788 platinum-based treatment NSCLC patients^4^. All genotyping was conducted by using Sequenom’s MassARRAY system (Sequenom, San Diego, California, USA). The correlation of GXG and GXE interactions in both discovery and validation stage were analyzed by using gPLINK (version 2.050, available http://zzz.bwh.harvard.edu/plink/gplink.shtml#down) and GMDR (available http://www.ssg.uab.edu/gmdr/) software^4,17,18^. Registration Number of ChiCTR-ROC-14005699 was acquired after meeting the clinical admission in the Chinese Clinical Trial Register. All patients provided written informed consent in compliance with the code of ethics of the World Medical Association (Declaration of Helsinki). The protocol used in this study was approved by the Ethics Committee of Xiangya School of Medicine, Central South University (Registration Number: CTXY-110008-1).^4^ The study design is summarized in Fig. 1.

### DNA extraction

5 ml fresh peripheral blood samples were obtained from patients using EDTA anticoagulant tubes. The plasma was removed from the whole blood by centrifuging at 2100 g for 10 min at room temperature. Wizard^®^ Genomic DNA Purification kits (Promega, Madison, WI, USA) were utilized for genomic DNA extraction. Primarily, blood cells were lysed and white blood cell nucleus was collected. Then, the nuclear membrane was broken and the nucleic acid was released after adding the nucleic acid lysis solution. The proteins in the mixed system were precipitated using protein precipitation solution and all the precipitation was discarded. Finally, isopropanol was added to the supernatant at an equal volume to precipitate the genomic DNA. Agarose gel electrophoresis was used for the quality control of these DNA samples, and the DNA samples that reached the quality control standard were stored in a −80 ultra-low temperature freezer before genotyping (QC of DNA, Data Citation 1). These methods are expanded versions of descriptions in our related work.^4^

### Genotyping

**Figure 2:**
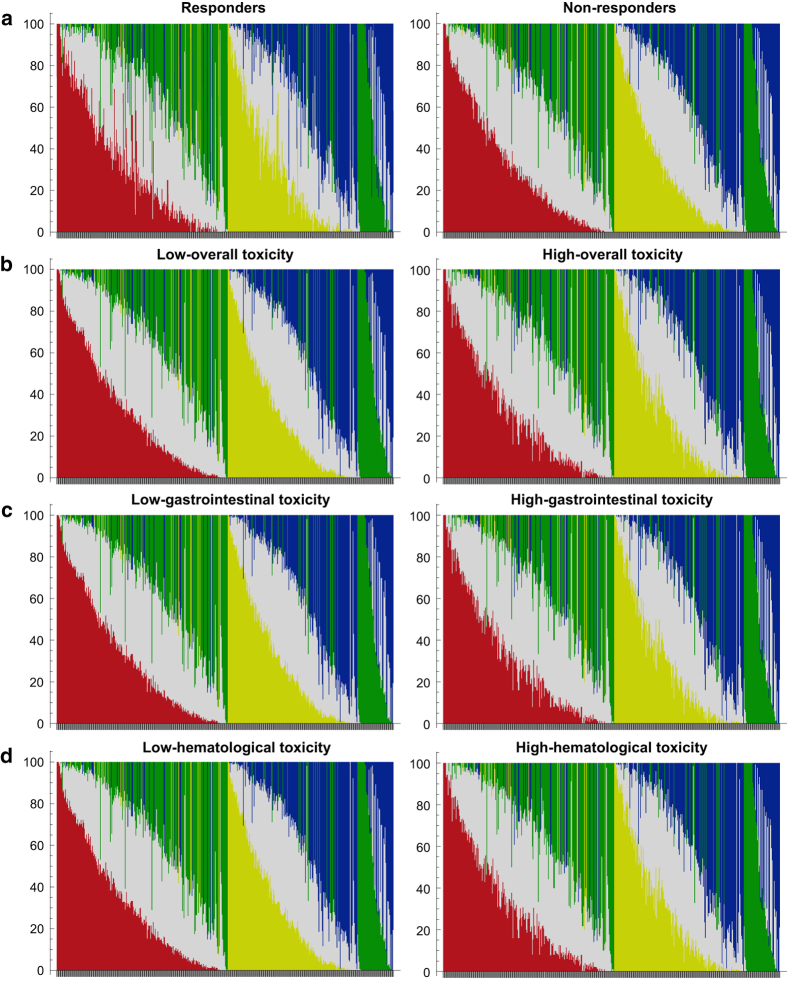
Landscape of genotyping in the discovery stage. (**a**) The genotyping results of 504 SNPs in the discovery stage of response analysis. (**b**) The genotyping results of 504 SNPs in the discovery stage of overall toxicity analysis. (**c**) The genotyping results of 504 SNPs in the discovery stage of gastrointestinal toxicity analysis. (**d**) The genotyping results of 504 SNPs in the discovery stage of hematological toxicity analysis. Each bar showed the percentage distribution of genotypes for each SNP. Red, yellow, blue, green and gray referred as the homozygous of T, A, C, G and heterozygous, respectively.

All genotyping experiments were conducted by Sequenom’s MassARRAY® system (Sequenom, San Diego, California, USA) as our related work briefly described.^4^ In detail, both amplification and genotyping primers were designed using MassARRAY Assay Design 3.1 software (SEQUENOM, San Diego, California, USA). At first, a 300–400 bp length sequence covering detected SNPs need to be prepared. It should be noted that the alleles of all other known SNPs must be replaced by “N”, while detected SNPs need to be provided as “wildtype allele/mutant allele”. Then, this sequence can be loaded by the software directly. In this study, the primers of 16 SNPs identified from discovery stage were provided in Table 1. To perform the multiple PCR, DNA samples were loaded into 384 well plates with reaction system containing dNTPs, primers, reaction buffer and DNA polymerase. After treating with shrimp alkaline phosphatase (SAP), the multiple PCR production can be subjected to single base extension using special mass-modified ddNTPs and primers. Finally, the productions purified with cationic resin were transferred into SpectroChip to perform genotyping. The raw data was interpreted by MassARRAY TYPER 4.0 software (pre-installed in the MassARRAY® System), and genotyping data was provided as both scatter plots, spectrums. To improve their reusability, additional accompanied quantitative *tsv* files and *txt* files were also provided. The landscapes of genotyping results were showed in Fig. 2. These methods are expanded versions of descriptions in our related work.^4^

### Data pre-process

All data should be pre-processed before analysis using gPLINK and GMDR. For gPLINK, *ped* and *map* files were the basic requirement, while *covar* files were required for adjusting the effect of covariate. In detail, *ped* file recorded the patients and genotyping information. The first six columns were family ID, individual ID, paternal ID, maternal ID, gender and phenotype, followed by the genotypes. *map* file recorded the information of SNPs. The four columns were located chromosome, identifier, genetic distance and base-pair positions. It should be noted that the order of “identifier” (from top to bottom) in the *Map* file and the order of “SNP 1 to N” (from left to right) in the *Ped* file must be the same. Otherwise, the calculated results would be incorrect. c*ovar* files recorded all covariates, for example, age, smoke stage and histology of patients. All covariates should be converted to binary form and each column recorded one covariate. In our study, all toxicity was evaluated according to the National Cancer Institute Common Toxicity Criteria 3.0 (NCI-CTC 3.0). In detail, we classified toxicity into hematologic toxicity (anemia, leukopenia, neutropenia and thrombocytopenia) and gastrointestinal toxicity. Each one was further scored from 0 to 4. Grade 0–2 was considered as low-toxicity and grade 3–4 was considered as high-toxicity. The response to chemotherapy was evaluated following the Response Evaluation Criteria in Solid Tumors (RECIST) guidelines. The curative effect was classified as complete response (CR), partial response (PR), stable disease (SD), and progressive disease (PD). We defined CR and PR as platinum-sensitive phenotypes, SD and PD as platinum-resistant phenotypes.^4^ The tumor stage and PS stage was evaluated according to the TNM Classification of Malignant Tumor (TNM) and Eastern Cooperative Oncology Group performance score (ECOG-PS) respectively. The smoking stage of these patients were defined as “yes” or “no”. The smoking stage of patients was defined as “no” only when the patient had never been reported smoked. Smoking stage of “yes” included both current and ever smokers. Available pack-years of patients were also provided in Genotyping Data (Genotyping Data, Data Citation 1).

For GMDR, *txt* files containing SNP information (for GXG analysis) or both SNP information and clinical information (for GXE analysis) were needed. Different SNPs and covariates should be listed in different columns. The phenotype of each patient needed to be provided in the last column. Headers, which describe the details of each column should be created for all genotypes, covariates and the phenotype in the first row. Subsequent analyses were all based on these files.

### Hardy-Weinberg equilibrium (HWE) and Linkage Disequilibrium (LD) test

Both HWE and LD test were performed using Haploview (version 4.2, available https://www.broadinstitute.org/haploview/haploview).^20^ It should be noted that *ped* and *map* files were also needed as described previously for this software.

### GXG and GXE interaction analysis

**Figure 3:**
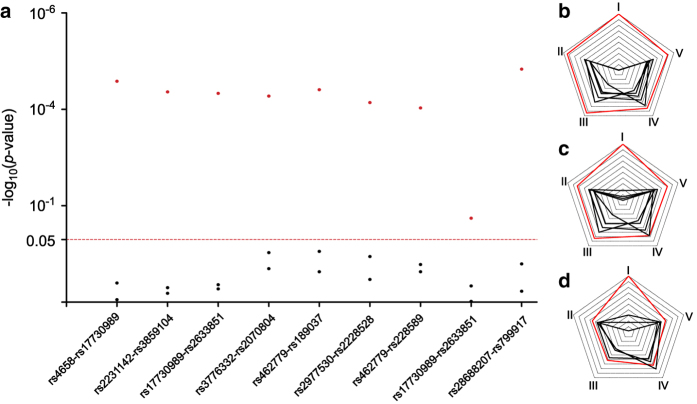
The performances of GXG and GXE analysis. (**a**) Correlation results of 16 SNPs which showed statistically significant association with platinum-based chemotherapy in the discovery stage analyzed by gPLINK. Red and black dot represented the *p*-value of paired and single SNP analysis, respectively. (**b**) Illustrative performances of GMDR models of rs3776332-rs2228528-rs228589 -rs2977549-Histology. (**c**) Illustrative performances of GMDR models of rs3776332-rs2228528-rs4658-Histology. (**d**) Illustrative performances of GMDR models of rs2228528-rs2929970-Histology. Red and black line represented the statistical significance (I), accuracy (II), sensitivity (III), specificity (IV) and precision (V) of model and univariate analysis.

The pre-processed data can be directly recognized by both gPLINK and GMDR. For gPLINK, the command we used for GXG interaction analysis was: plink --map “xxx.map” --ped “xxx.ped” --epistasis --epi1 0.05 --covar “xxx.txt” --covar-number 3 --out “xxx” --gplink. The “xxx” referred to the filename. The command “--epistasis” was utilized for epistasis analysis, “--epi1 0.05” was used to define 0.05 as the threshold value of significant statistical difference. “--covar-number 3” was utilized for adjusting the covariate in the third column in *covar* file. “-- out” was utilized for results output. “plink” and “--gplink” were necessary for commands to be recognized by this software. For GMDR, pre-processed genotypes files were loaded using “Load Marker” in the “Analysis” Tab. To define the number of dimensions included in the final model, “Marker Count Range” function in the “Configuration” Tab could be used. Then, GXG and GXE analysis was completed by clicking “Run Analysis” button in the “Analysis” Tab. Our results showed that GXG and GXE models incorporating multiple variates performed better than univariate analysis, which were showed in Fig. 3.

## Data records

### Genotype

Scatter plots in both discovery and validation stages was provided (Scatter Plots, Data Citation 1). In addition, each spectral data of 788 patients was indicated in Spectrums Data (Spectrums Data, Data Citation 1). Accompanied additional *tsv* files for Scatter Plots and Spectrums Data were also provided. For Scatter Plots (Scatter Plots, Data Citation 1), *tsv* files contained patients’ ID and the MASSArray quantity of major and minor allele for markers of each patient. For Spectrums Data (Spectrums Data, Data Citation 1), *tsv* files contained intensity at specific mass-to-charge value in spectrums. A “Master Mass List” file was also provided as an accompaniment for *tsv* files of Spectrums Data (Spectrums Data, Data Citation 1). This “Master Mass List” file showed the spectrum files’ name and the m/z value from which each scatter plot value was retrieved for each marker. Each patient’s genotype and their clinical characteristics were provided as *txt* files in Genotyping Data (Genotyping Data, Data Citation 1). Information about all designed primers for multiple PCR and single base extended were provided in Supplementary Table S1.

### Pre-processed data files

In the previous study, all pre-processed data files were generated from Genotyping Data (Genotyping Data, Data Citation 1). *Ped*, *map* and *txt* files for gPLINK and GMDR were all presented based on phenotypes of sensitivity, overall toxicity, hematological toxicity and gastrointestinal toxicity. All clinical variates showed significant association with given phenotype should be adjusted, therefore, we adjusted different clinical variates for different phenotypes. For example, in the discovery stage, the histology was adjusted when we performed GXG analysis for overall toxicity, while the age was adjusted when we performed GXG analysis for gastrointestinal toxicity.

To make this process clear, we provided all files utilized in GXG and GXE analysis for overall toxicity. For gPLINK, Ped.ped and Map.map were used for GXG interaction analysis. Covar.txt was used for covariates adjusting. For GMDR, GXG.txt and GXE.txt were used for GXG and GXE interaction analysis. These files can be found in Pre-processed Data Files (Pre-processed Data Files, Data Citation 1).

In Data Citation files, the “1” referred low-toxicity, platinum-sensitive phenotypes, male, age <60, non-smokers, adenocarcinoma cancer, stage III and PS stage = 0 respectively; In the contrary, the “2” referred high-toxicity, platinum-resistant phenotypes, female, age ≥60, smokers, squamous cell cancer, stage IV and PS stage > 0 respectively. The “0” referred the empty value.

### Results of interaction analysis

For gPLINK, result files with an extension of *.epi.cc* were generated by software automatically. For GMDR, results of each model could be outputted separately and opened as *txt* files. All raw data generated by gPLINK and GMDR was packaged in Raw Analysis Data (Raw Analysis Data, Data Citation 1).

## Technical Validation

### Quality control of genomic DNA

Genomic DNA concentration was measured in NanoDrop 2000c spectrophotometers (Thermo Fisher, Wilmington, Delaware, USA). The ratios of absorbance in the length of 260 nm/280 nm were recorded, 1.8–2.0 was qualified for genotyping. In addition, integrality of genomic DNA was examined in agarose gels according to the location of bands.^19^ All low-quality samples were removed in this step.

### Quality control of MassARRAY genotyping

Productions of multiple PCR were observed in agarose gel. Briefly, 1 μl PCR productions were diluted in 1 μl 2× loading buffer and analyzed using 1% agarose gel. Bands’ specificity was used to evaluate PCR quality. TYPER 4.0 software was utilized to evaluated the quality of all SNPs. Genotypes with low signal-to-noise ratio or in the interface of two genotype were identified as low probability SNP calls.

## Usage Notes

### DNA extraction

When the genomic DNA was extracted from frozen blood, washing the whole blood with red cell lysed agent twice may be essential to ensure that red cells were full lysed. To obtain enough high quality genomic DNA, nuclei should be lysed sufficiently and protein should be precipitated thoroughly as well. To guarantee the genotyping accuracy, quality of each patient’s genomic DNA should be carefully examined.

### MassARRAY genotyping

Primer design was the most important step for genotyping. To get reliable results from mass spectrometer, mass of extended primers should be between 4.5 to 9.0 KDa. A difference of 30 Da among different primers was necessary for distinguishing various extended productions. It was noted that the concentration of primer should be diluted into 4 different levels according to their un-extended mass. Primers powder should be centrifuged at 3,000 rpm for 3 min before they were dissolved to avoid inaccurate final concentration. All steps should be performed carefully on ice. 384 well plates should be sealed in some steps to prevent the evaporation of productions. When prepare the mix reagents for multiple PCR, SAP reaction and primer extension, using a 30% excess volume of each reagent to ensure its sufficiency for the next step is important.

### Data analysis

Correct data format was the fundamental requirement for successfully running software. For gPLINK, the missing data should be recorded as value “0 0”, while that was “..” in GMDR. All covariates should be converted to binary format which helps them to be recognized by software easier. If errors occurred in the process, the format and the locations of these files should be checked carefully to ensure that these files could be recognized by software.

## Additional information

**How to cite this article**: Wang, L. Y. *et al*. Gene-gene and gene-environment interaction data for platinum-based chemotherapy in non-small cell lung cancer. *Sci. Data*. 5:180284 doi: 10.1038/sdata.2018.284 (2018).

**Publisher’s note**: Springer Nature remains neutral with regard to jurisdictional claims in published maps and institutional affiliations.

## Supplementary Material



Supplementary Table S1

## Figures and Tables

**Table 1:** Primers of 16 SNPs for MassARRAY identified from discovery stage.

Chr.	SNP	Location	Gene	Primer-EP	Primer-F	Primer-R
1	rs4658	43392250	SLC2A1	TCCAGGCCAGCAGAA	ACGTTGGATGAAAGCTTCTATCCCAGGAGG	ACGTTGGATGAATCCTAATGGAGCCTGACC
2	rs17730989	198362524	HSPD1	ACCATCAAGGCAAGTAG	ACGTTGGATGTATGTTGCGTGAACCTGGAA	ACGTTGGATGGTGACTTGTTTTAAAATCCG
3	rs2633851	4403817	SUMF1	GTGGACTGGGGAAGACT	ACGTTGGATGGCCCACTATGGACTGACAAC	ACGTTGGATGAGAAAAGCCCAATGTAGGTC
4	rs2231142	89052323	ABCG2	TTTAGAAGAGCTGCTGAGAACT	ACGTTGGATGTGATGTTGTGATGGGCACTC	ACGTTGGATGCGTCATAGTTGTTGCAAGCC
5	rs3776332	142441514	ARHGAP26	TTATGTCACATCTCATT GGTC	ACGTTGGATGCCCACAAGTGGCTCAGATAA	ACGTTGGATGTCCAGCACACTTTATGTCAC
6	rs28688207	32628660	HLA-DQB1	GAGGCCCTTGAGGTC	ACGTTGGATGAATATTACCTGCTGGTGGAG	ACGTTGGATGTGAGAGAGTGGCTGTTTGTG
6	rs462779	111695887	REV3L	TTATTTTTTCATCCTTAAGTGTT	ACGTTGGATGACTTAACCTCAGCACCAGAC	ACGTTGGATGAATGAGAAAGGTACATCGAG
7	rs2070804	75933712	HSPB1	CCGAAACCTACACCAGTGTACCC	ACGTTGGATGCAGGAGTCATCTTTGCTCAG	ACGTTGGATGATGTGAGTCAGCCTGTGTCC
8	rs2977530	134215112	WISP1	CCTCTGAGTCAGCCA	ACGTTGGATGAGGTGTTGGGAAAAGAGGTG	ACGTTGGATGTCCCCACGCTTGTTTCAAAG
8	rs2929970	134241137	WISP1	AGGAAGATGGAGGTTTACC	ACGTTGGATGGCTTCAACCTCTTCAGCTTT	ACGTTGGATGTTCTGGTAGGAAGATGGAGG
8	rs2977549	134242033	WISP1	ATATTAAATGTCTCTTTTGCTAAG	ACGTTGGATGCATACATATGCATTTCTTTG	ACGTTGGATGCCAAAGCTACATGAAAATAG
10	rs2228528	50732280	ERCC6	CTTCAGCTCATAGTCAGTA	ACGTTGGATGAGGAAGATGACGAGGTGGAG	ACGTTGGATGGCAGAGGCTTCAGCTCATAG
11	rs228589	108093208	NPAT	GGTCCAATAACCCTCC	ACGTTGGATGCTTGTATTGGGTAAGCGCGG	ACGTTGGATGTTTGGCCTCAAAGGTCCTTC
11	rs189037	108093833	ATM	TCTCGCCTCCTCCCG	ACGTTGGATGGCTAACGGAGAAAAGAAGCC	ACGTTGGATGGTCAAAGTAGTATCAACCGC
16	rs3859104	55895249	CES5A	TTCTATGTTCCAAATTCAAATAAATA	ACGTTGGATGCTTCGAACGGAGAGATGAAC	ACGTTGGATGCAAGTACCTCAATCCTCCTC
17	rs799917	41244936	BRCA1	AAGCGCCAGTCATTTGCTC	ACGTTGGATGAAGGTTTCAAAGCGCCAGTC	ACGTTGGATGAGAGTGGGCAGAGAATGTTG
