# Supplementary material for: Gene-gene and gene-environment interaction data for platinum-based chemotherapy in non-small cell lung cancer
Source: Sci Data. 2018 Dec 11;5:180284. doi: 10.1038/sdata.2018.284 (PMC6289114; doi:10.1038/sdata.2018.284)
Supplement: Supplementary Table S1 [file sdata2018284-s2.docx]

**Table S1:** Primers for multiple PCR and single base extended.

| Chr. | SNP | Location | Gene | Primer-EP | Primer-F | Primer-R |
| --- | --- | --- | --- | --- | --- | --- |
| 2 | rs17730989 | 198362524 | HSPD1 | ACCATCAAGGCAAGTAG | ACGTTGGATGTATGTTGCGTGAACCTGGAA | ACGTTGGATGGTGACTTGTTTTAAAATCCG |
| 7 | rs2070804 | 75933712 | HSPB1 | ACCTACACCAGTGTACCC | ACGTTGGATGCAGGAGTCATCTTTGCTCAG | ACGTTGGATGATGTGAGTCAGCCTGTGTCC |
| 1 | rs4658 | 43392250 | SLC2A1 | TCCAGGCCAGCAGAA | ACGTTGGATGAAAGCTTCTATCCCAGGAGG | ACGTTGGATGAATCCTAATGGAGCCTGACC |
| 6 | rs707939 | 31726688 | MSH5 | AAGATTGGGTGTAGCCTTCAGATGT | ACGTTGGATGGCCCCACAAGTTTTCTTATC | ACGTTGGATGGAGAGTGGGTGTAGCCTTC |
| 7 | rs3813517 | 6448532 | DAGLB | TGTTGTTGGAGTGTCTT | ACGTTGGATGCCAAAAAAGCAGAGACCCAC | ACGTTGGATGCCCCTTAAGGTTCTGTACTG |
| 2 | rs10191478 | 47645249 | MSH2 | AGACACGTATACAATGTATAATACTTAA | ACGTTGGATGTGAGGTGATGGATATCTCAG | ACGTTGGATGCATAATTGTACACATTTGGG |
| 19 | rs1046282 | 45910672 | PPP1R13L | CAGTAGAAATACTAACAAAGGGC | ACGTTGGATGAGTGTCCTCAGAAAGCAGGG | ACGTTGGATGCTGCACACTCCAAGTTTAAC |
| 2 | rs1051677 | 217070248 | XRCC5 | AGGTCTGCAACTGTCACTGATAT | ACGTTGGATGACATGCTTTGAAGTTTCTGG | ACGTTGGATGTAATCACATCACAAGGGCTG |
| 2 | rs1051685 | 217070376 | XRCC5 | TCCCGATCCTCCAACAGCTGTCACA | ACGTTGGATGGCAAATGCTACTGCTTGCTC | ACGTTGGATGCTTAACCCTTTCCAGAGTCC |
| 1 | rs1053513 | 229652587 | ABCB10 | CTCCTGTTTACTAATTCAAAGAACACTA | ACGTTGGATGCATGAAAAGCATGGAATAT | ACGTTGGATGGATGTACTGTTTACTAATTC |
| 16 | rs1057451 | 29833488 | PAGR1 | TAAAATTGATGAAGATCAGGGGT | ACGTTGGATGAGAACCGCCTAGATCAGAAG | ACGTTGGATGGCTCAGTCCTATGATTAGGC |
| 3 | rs10849 | 37095070 | LRRFIP2 | GCAGCTGGGGAAAAA | ACGTTGGATGAGGGTAGAACCAGTCCATGC | ACGTTGGATGAGTTACTGAGGCAGCTGGG |
| 12 | rs10875989 | 50351075 | AQP2 | ACTATTCTTACCCTGAATGTGTGCCC | ACGTTGGATGAAGGAGAGAATGGATAGGGC | ACGTTGGATGACCTCTTCTTACCCTGAATG |
| 11 | rs10896607 | 57106459 | P2RX3 | GGATCTCCATCTCCACCCT | ACGTTGGATGAATTCGAAGATGTGGGAGGG | ACGTTGGATGAGGGCTCAGGAGCTCCATCT |
| 7 | rs10951983 | 6446027 | RAC1 | ACGATCAGAATGTATACCCATCGTTA | ACGTTGGATGTTTCAAAGATGCAGGGGCTC | ACGTTGGATGAAATCACCCGCGTGTTTCTC |
| 17 | rs1131636 | 1801189 | RPA1 | AAGAGCAGCATTAATTGAAGTGA | ACGTTGGATGAAATCCGAGCGGCTACAAAG | ACGTTGGATGAAAGTACGCACTCACCAAGC |
| 1 | rs1146642 | 76261434 | MSH4 | GAGAGGAGCTGTGATTGCACTACTA | ACGTTGGATGACAGGGTCTTCCTTTCTTCC | ACGTTGGATGTTGAGCCCAGGAGTTTAAGG |
| 6 | rs1150793 | 31717696 | MSH5 | CCCGCTGCAATATGT | ACGTTGGATGCTGATTACCTGGAGATGACC | ACGTTGGATGAATCCTTCCCCTACCTCACC |
| 19 | rs117128015 | 45910903 | PPP1R13L | CCGTCTGACCACCAATTATAATTACA | ACGTTGGATGGCTTGGTGGAACATGTTCTG | ACGTTGGATGAGGTTCATGCTTATGGCCTG |
| 10 | rs12220909 | 14425221 | MIR4293 | CGAAGGCTGTTCCTGTCA | ACGTTGGATGGCTGGTGACATTGCTAATTC | ACGTTGGATGGGGAGAGGGGAAATCCTATT |
| 7 | rs12536544 | 6435901 | RAC1 | TTTCTTCCCCGTCCT | ACGTTGGATGAAGGAAACTGAGAGACCCTG | ACGTTGGATGCTGGGAGGCTCTATTTCTTC |
| 17 | rs12727 | 1801065 | RPA1 | ACCATCTGACTCAGTCTATTAAA | ACGTTGGATGTATCTGAAGAGTATCCTTCC | ACGTTGGATGTTATCAGCGGTCCTCAGAAG |
| 10 | rs12778366 | 69643079 | SIRT1 | TCATCTGGTCACCACT | ACGTTGGATGTAAGGCTTCTAGGACTGGAG | ACGTTGGATGCTAAGGTCCTATCTACATCC |
| 19 | rs12983892 | 45913309 | CD3EAP | CCGCCCCTGCCCAGCTAA | ACGTTGGATGTAACGTGGTGAAACCCCATC | ACGTTGGATGTGAGTAGCTGGGACTACAGG |
| 19 | rs12984195 | 45913460 | ERCC1 | CCAGGAGACTGTCTCA | ACGTTGGATGTTATAGGTGTGAGCCACTGC | ACGTTGGATGAAGATCACACCACTGCACTC |
| 2 | rs12999145 | 47686686 | MSH2 | ACTGTATACCTAGAAAAACCA | ACGTTGGATGGTCTGTATTTCACCCTTGTTC | ACGTTGGATGGGGCAACAAGAGCAAAACTG |
| 2 | rs13019654 | 47689217 | MSH2 | GGGAGAGGTGACATACTGAGACT | ACGTTGGATGCTCTTCTCTTCTCTGTCTTT | ACGTTGGATGAGGCAGAGGTTGCAATGAGC |
| 2 | rs13386066 | 198371687 | HSPE1_MOB4 | CAAGCCATAGCCTTTGAACAGA | ACGTTGGATGAATCGTTCCAAGGACATGTG | ACGTTGGATGTGGGAAAGCCATAGCCTTTG |
| 1 | rs1385129 | 43408966 | SLC2A1 | ATCACTGCTCCTCCCAC | ACGTTGGATGGGTTTGTTTCTCCGCAGAAG | ACGTTGGATGTGTTGTAGCCAAACTGCAGG |
| 4 | rs1448784 | 89012320 | ABCG2 | AACATCAATTCAGGTCAAGAAA | ACGTTGGATGAGTAGTGACTGGGAGAATGG | ACGTTGGATGTGTGCAACCATCAATTCAGG |
| 15 | rs1516400 | 58429264 | AQP9 | GAATTCATCAGGAATTAAAGCCAAA | ACGTTGGATGTGCAGAACCAGGACAAAACC | ACGTTGGATGGGTGTGGTCATAGGTAACTC |
| 3 | rs1540354 | 37044489 | MLH1 | CACACAGAAATCCCTCAATAAATCT | ACGTTGGATGTCGCCTAGCTGTAGGTTATC | ACGTTGGATGCTGGTACACAGAAATCCCTC |
| 15 | rs1554203 | 58429547 | AQP9 | ATCATAAACTAGGCCTGCCTTGCT | ACGTTGGATGCCTTGTTTTTCAAAACTGGC | ACGTTGGATGTAATCAGAAACTAGGCCTGC |
| 5 | rs1650665 | 79962578 | MSH3 | TCTGTTTGATGGCATGGATAGA | ACGTTGGATGTCACTCAGTAGCTTTTTGCC | ACGTTGGATGCACGTAGAAGCAGTGCCATT |
| 7 | rs17064 | 87133470 | ABCB1 | TGTTAAACAGATACCTCTTCA | ACGTTGGATGCAAAGTTAAAAGCAAACAC | ACGTTGGATGGACTCTGAACTTGACTGAGG |
| 19 | rs172731 | 11446951 | RAB3D | AGAGGTCCCAGGTGCTGGTGACCAAC | ACGTTGGATGTCCCCTCTAAGATGGTACCC | ACGTTGGATGAAAAGAAAGGTCCCAGGTGC |
| 17 | rs17292622 | 1802418 | RPA1 | CCGTGGATGTGATTGG | ACGTTGGATGTGTTAGAAACAGCCTCCTCG | ACGTTGGATGTGATGAAACCAGCACGTCTC |
| X | rs17330644 | 123043876 | XIAP | GAAGAAGTGGTGACTTGAAATCCTA | ACGTTGGATGTCCCAAGAGTTCTCAGTGTC | ACGTTGGATGGTAAAATAAGTGGTGACTTG |
| 17 | rs17339382 | 1801872 | RPA1 | GCCCATATCCAAAAGCCTC | ACGTTGGATGTGTTCATCACTGCACATGCC | ACGTTGGATGGAGACAGACAGGACACCATC |
| 17 | rs17339395 | 1802501 | RPA1 | CCGCGCCAGTTTCTGATGCTCCTCTG | ACGTTGGATGTCGAGGAGGCTGTTTCTAAC | ACGTTGGATGAGTGGAGCCAGTTTCTGATG |
| 17 | rs17734 | 1801144 | RPA1 | TCGGATTTCCTTAGGGGAC | ACGTTGGATGATTGTCTCTTCTGAGGACCG | ACGTTGGATGAAGAAACGCTTTGTAGCCGC |
| 1 | rs17739 | 223967953 | TP53BP2 | GGTTTTTGAAATATTGAAACTAAGCTAC | ACGTTGGATGGCCTACAAACAAGATTCTAC | ACGTTGGATGTATGAGTTTTTGTAGCATC |
| 15 | rs1867380 | 58476281 | AQP9 | CTAGCCTGACTCAGTCTTTAAG | ACGTTGGATGGCCACTACATGATGACACTG | ACGTTGGATGTGAAATCCACCATCCAGAGC |
| 6 | rs1869641 | 99277867 | POU3F2 | GTCCTTCTTCAAGACCTTTTATTACA | ACGTTGGATGGCAAATTCCTAAATGTGCATC | ACGTTGGATGCCAAGCATATGACGTAGGAG |
| 6 | rs1883306 | 99279449 | POU3F2 | TCTGGTTTTAGGTCCAGAC | ACGTTGGATGAAAGAATGGGCCCCAGAGC | ACGTTGGATGTGAGCTCTCGCTGGTTTTAG |
| 6 | rs195854 | 99289475 | POU3F2 | AAGCTTAAGTTGCCTTTCTTTTTTT | ACGTTGGATGGATGCACTTTTACTGGTGTA | ACGTTGGATGCCTAGAATTCTGATTGTTGG |
| 6 | rs195862 | 99281809 | POU3F2 | CTGTGTGCGGACACTTC | ACGTTGGATGAAAAGGAAGAGAGCCTGTGC | ACGTTGGATGACAAGTGTCTCTTGCTCCAG |
| 2 | rs1981929 | 47672569 | MSH2 | ATCCAAAAGGTACAAATAGTACAG | ACGTTGGATGCACCTGAGTAAATAGTAAC | ACGTTGGATGGACTGAGATAAACAAGCATC |
| 7 | rs2009836 | 75931160 | HSPB1 | CCCAGACTGCTTTATTTTTGTA | ACGTTGGATGCTACAGAGCAAAACCCTGTC | ACGTTGGATGCCAGCCCAGACTGCTTTATT |
| 2 | rs2020910 | 48030692 | MSH6 | ATCCTTGCATTACGAAGAC | ACGTTGGATGTTTTCCTGCTCCTCTTCCTC | ACGTTGGATGTCACGCCATCCTTGCATTAC |
| 15 | rs2077737 | 58478950 | AQP9 | CCACCCCTACCCCCA | ACGTTGGATGCTCCACAAAGGTAAATCCAC | ACGTTGGATGTGCTTTTGTTCCAGGTGCTC |
| X | rs2227291 | 77268502 | ATP7A | CCTACTCTTTGATTATTCTTCTA | ACGTTGGATGAAAATTAGCCGGGCGTGTTG | ACGTTGGATGCTGGGTTCAAGCAATTCTCC |
| 6 | rs2293852 | 31699980 | DDAH2 | TACAGGTGTGTGGTTATT | ACGTTGGATGACTCCAGTGGTCATCCTCTC | ACGTTGGATGACTGCAACCTGTTGCCAAAG |
| 2 | rs2348244 | 48019485 | MSH6 | TTCCCAGAATCACACTC | ACGTTGGATGAGACAAAATGCCCCCAATCC | ACGTTGGATGCCCAGGTAACTGGGAAATTC |
| 2 | rs2440 | 217070766 | XRCC5 | AGGTAGAAATGGGAAAGA | ACGTTGGATGGTTCTCTACTTGCAAGCCTC | ACGTTGGATGGCTGACTTAGGTAGAAATGG |
| 6 | rs2444933 | 99278428 | POU3F2 | TTGCGGTAGCAGCTGGTTTAAACCTA | ACGTTGGATGAACATGATCCTCTAAGGTGG | ACGTTGGATGTGGAGGTAGCAGCTGGTTTA |
| 5 | rs245340 | 80161500 | MSH3 | GGAAAGGCTGGTGGCTTAAAACAATTA | ACGTTGGATGGCCATAAGTAATACAGTAGAC | ACGTTGGATGAAACCACCACAGGACTACAG |
| 5 | rs245346 | 80156059 | MSH3 | GGGATTCACTGGTTCCTTCTTCA | ACGTTGGATGAAGGAAGGAAAATGACAGGG | ACGTTGGATGTTTTCGCCTTCACTGGTTCC |
| 2 | rs2605039 | 198362851 | HSPE1_MOB4 | AAAATTGTAACTTTGTACTCATTA | ACGTTGGATGTGGAGGAGAGAATGGGAAGC | ACGTTGGATGCCACCATCCATCTCCAAAAC |
| 5 | rs26778 | 80035750 | MSH3 | ATAGCTATAATGAAATTTAGGTAACA | ACGTTGGATGTCTAAAGTTCTGGGTGAGGG | ACGTTGGATGCTAACCACCCTTCTCATAGC |
| 5 | rs26784 | 80049892 | MSH3 | CAAATAATTTTTTAAATGCTAAGAAGAA | ACGTTGGATGGCCAGAGATGGTTTTTGTTG | ACGTTGGATGTGTGAAGATATTGCCTGTTC |
| 2 | rs2710163 | 48035673 | MSH6 | GGTCTGGAGCCACAAAGAATTT | ACGTTGGATGACCTTTGGCTTTAAGAGGAC | ACGTTGGATGTCAGAATTAATCTGGGCTGG |
| X | rs28382740 | 123041043 | XIAP | AAATTTTTATGTCTTAATCTAACTCTAT | ACGTTGGATGCATCACACATTCAATCAGGG | ACGTTGGATGCACAGTCATTACTTTCAAGC |
| X | rs28382746 | 123044642 | XIAP | GGAAGGAGAATGGTGTGAACC | ACGTTGGATGAGAGTCTTGCTCTGTTGCCC | ACGTTGGATGCTGAGGCAGGAGAATGGTG |
| X | rs28382751 | 123045109 | XIAP | CCCGCGACCTCCCGACCTCAG | ACGTTGGATGTAATCCCAGCACTTTGGGAG | ACGTTGGATGTTTCTCCATGTTGGTCAGGC |
| X | rs28382752 | 123045219 | XIAP | CCTGTAATCCCAGCTACT | ACGTTGGATGGGGTTCAAGCGATTCTCCTG | ACGTTGGATGACAAAATTAGCTGGGCGTGG |
| 15 | rs28599926 | 22513271 | LOC101928039 | GGGTAAAGAAAAGTTAGCCGGGC | ACGTTGGATGAAAGTAGCTGGGACCACAGG | ACGTTGGATGGGTGTAAGAACCTGTATCTAC |
| 7 | rs2868370 | 75930800 | HSPB1 | CTCATCTGGTGTGGTTGTTAGG | ACGTTGGATGTGTGAATTCAGGCAGTCTGG | ACGTTGGATGGCAATCTAGTAATCTGGTGTG |
| 7 | rs2868371 | 75930759 | HSPB1 | GCATCTGCTTAGTGATGGAA | ACGTTGGATGTGTTAGGCGTGTGGACTTTG | ACGTTGGATGGCACAAGTTCATCTGCTTAG |
| 7 | rs2961047 | 75935056 | HSPB1 | GAACTGCATGCTCCGCCT | ACGTTGGATGCTGAGGCTGTAGCAAGATAC | ACGTTGGATGAGTGCAGTGGCACGATCTC |
| 12 | rs296766 | 50350953 | AQP2 | ACCCTTACCTCCTTTCG | ACGTTGGATGCAGGACTCTCCTTTGCTTTG | ACGTTGGATGAGTCTGATGCTCAACATCCC |
| 5 | rs3088225 | 132387260 | HSPA4 | GTGCCTTCTGCTCTT | ACGTTGGATGTTCCAATGGCGGCCTTCTC | ACGTTGGATGATGAGACGCAAGCTGATCTG |
| 6 | rs3115672 | 31727897 | MSH5 | GGAACTCTGTGCCCGAAC | ACGTTGGATGTGACTTTGACCCTCCCTTTG | ACGTTGGATGTCCTCTGATGGAACTCTGTG |
| 6 | rs3117572 | 31717692 | MSH5 | ACCTCCCGCTGCAAT | ACGTTGGATGCTGATTACCTGGAGATGACC | ACGTTGGATGAATCCTTCCCCTACCTCACC |
| 2 | rs3136329 | 48024876 | MSH6 | TGAACCTGCTACTGTGCT | ACGTTGGATGGAAGACAATAACATCTTGAGG | ACGTTGGATGTCAGCGATTTTCTCCACAGG |
| 9 | rs3176751 | 100437518 | XPA | CAATCACAGACATGACATT | ACGTTGGATGTTCAGTGAAGGTCACCTGGC | ACGTTGGATGTAGCACTCAGCTCCCATCTC |
| 9 | rs3176752 | 100437487 | XPA | CTCAGCTCCCATCTCT | ACGTTGGATGATGTCATGTCTGTGATTGCC | ACGTTGGATGTAGCTGACCTACCACTTCTG |
| 14 | rs3212117 | 104164687 | XRCC3 | TGGTGGAGTAAGGACTGCCCT | ACGTTGGATGTCAGACCCCCTCAGCCTTG | ACGTTGGATGACTGTGGCCTGAGTGGTGG |
| 14 | rs3212118 | 104164634 | XRCC3 | CCCCCAGAGGTGGGTGAGGAGGAGCC | ACGTTGGATGACACAGGGCAGTCCTTACTC | ACGTTGGATGAAGGACCCAGAGGTGGGTGA |
| 14 | rs3212121 | 104164522 | XRCC3 | CTCATGGACCCTGACACTGGAGGT | ACGTTGGATGTGGTACAAGGTGGAGAAAGC | ACGTTGGATGTACTAGAGACAAGGCAGTCC |
| 2 | rs3732190 | 48037593 | FBXO11 | CCCTCGTAGTCTGTTATATGCATTAGC | ACGTTGGATGTCATTTTCTTCAAGGAGACC | ACGTTGGATGCCACCAAAAAGGTTAGTAGTC |
| 1 | rs3738514 | 43426591 | SLC2A1 | AGGCAGAGGAGACAA | ACGTTGGATGTCACACCAGCAACTCACATC | ACGTTGGATGTGCTTTTTGGCATGGTGAGG |
| 10 | rs3740051 | 69643959 | SIRT1 | ACTGTTGCCTCTCTTCCTACTT | ACGTTGGATGAGCTCCCTGAAATACGTTGG | ACGTTGGATGAAAGGAGCCGCCTCCTTTTG |
| 17 | rs3744766 | 1800949 | RPA1 | AGAAAGAGAGAGCCTTC | ACGTTGGATGTGAGAAGCGGTCATGAGCAC | ACGTTGGATGTACCAACTATGTAGCACCTC |
| 17 | rs3744767 | 1800932 | RPA1 | ATCACCTGGGGATTTTAGTAAG | ACGTTGGATGCACCTCTAGAAAGAGAGAGC | ACGTTGGATGTGAGAAGCGGTCATGAGCAC |
| 17 | rs3744768 | 1800886 | RPA1 | CTTCTCAGCTCGCCC | ACGTTGGATGTTTGCTTCTCCAGTGGTGAC | ACGTTGGATGTGCTCATGACCGCTTCTCAG |
| 17 | rs3744769 | 1800849 | RPA1 | GCAGTGGTGACCACCC | ACGTTGGATGAAGAACCCAAGTTGTGAGCG | ACGTTGGATGCCTAACTGCAGACCATGAAG |
| 10 | rs3758391 | 69643342 | SIRT1 | CAAGGACCCATATAACCC | ACGTTGGATGGCCATAACAAACACTGGCTC | ACGTTGGATGGCACACTGTGACTCCATATC |
| 12 | rs3759125 | 50343608 | AQP2 | GCCCCAGCTTCTGGGACCCCAACA | ACGTTGGATGTGCACTTTGAGGAGAACAGG | ACGTTGGATGTTTCTAGGACCTGTCACAGC |
| 12 | rs3759126 | 50343862 | AQP2 | CCCCACTTGGTGATGGAGTCAACC | ACGTTGGATGGAATGGACACCCTCAGCTTC | ACGTTGGATGAAACAGCAGCTCCTAGAGCC |
| 1 | rs3806162 | 76261995 | MSH4 | GGGGACTGCAAAGCTGGAG | ACGTTGGATGCTAGTACTGTGGTTTCTAAC | ACGTTGGATGTAAAGGTCACCACTGCAAAG |
| 1 | rs3806400 | 43426378 | SLC2A1 | GCTGTCTACACCACCTCATC | ACGTTGGATGAAAGACCAGAGGAGATGGAG | ACGTTGGATGAGACCAAGTCCTGCTGACTC |
| 3 | rs3811699 | 49396360 | GPX1 | CTCATTGGGGGTAGGGTGA | ACGTTGGATGAAGGAAACGCTGCCGGAGTC | ACGTTGGATGTTACCCTGTACCGCATTCTG |
| 5 | rs3816729 | 80040532 | MSH3 | AAATAAAATGTTCTCCAATTTAAAAC | ACGTTGGATGAGGCATTTTAAAACCAAGG | ACGTTGGATGGCCAGTCTATTGCTCAGTTC |
| 1 | rs3820589 | 43426044 | SLC2A1 | GAATTGAGACACAGTGAAGGG | ACGTTGGATGAAGGGACCCTCAAGACTTTG | ACGTTGGATGAGACCAGTGCCTTACTTGAG |
| 6 | rs3823036 | 99284532 | POU3F2 | CCCCCATGCTGCTGTGTTTATTTATTG | ACGTTGGATGTTTTTCCTCTTTCCTGATGG | ACGTTGGATGTGCAGCTTCCTGATGCTTAG |
| 5 | rs394592 | 80172317 | MSH3 | CTCTGGGACTACAGGTGTG | ACGTTGGATGCGAAAAAATTAGCTGGGCAT | ACGTTGGATGGTTCAAGAGATTCTCCTGCC |
| 6 | rs409558 | 31708147 | MSH5 | CCTGTAGCAGAAGTACTTAG | ACGTTGGATGGAAGCAACGATTCACAGAGG | ACGTTGGATGGCAGCCCTGTAGCAGAAGT |
| 2 | rs4608577 | 47703984 | MSH2 | CCTCAATCTCCACTACCATGTT | ACGTTGGATGAAGGACCATTCCAGGAAGTG | ACGTTGGATGAGCTACTGGTACGAGTTCAC |
| 5 | rs4616886 | 132423764 | HSPA4 | TGATGGCTAGCGTGT | ACGTTGGATGGTACAGGTCCTGAATTTCTC | ACGTTGGATGCCTCTTATTTTCCTGATGGC |
| 12 | rs461872 | 50345206 | LOC101927318 | GCTCTTCCTGCACGA | ACGTTGGATGAAAGCAGGATGCAGACAGAC | ACGTTGGATGCCAACAGAAACCTGGATGAG |
| 22 | rs4630 | 24376322 | GSTT1 | GACTTGGGCAAGTCTTA | ACGTTGGATGTTTCTAATGGCCCATGGGAG | ACGTTGGATGTGAGGTGCTCTGGGACTTG |
| 7 | rs4720672 | 6443839 | RAC1 | GCACACACACCCCGAT | ACGTTGGATGTTGGACCTGTCGAACAGTGG | ACGTTGGATGAACATCTCCATCTTCAGCCC |
| 10 | rs4746720 | 69676830 | SIRT1 | GTTTAAAAATAATTGTGTTAAAGAATCA | ACGTTGGATGGTACTCAAAATCTGTTACGC | ACGTTGGATGGCCACAGTTTTGGAAAATGC |
| 16 | rs4788184 | 29830426 | MVP | ACCGGGTTCAAGCGATTCT | ACGTTGGATGCCTGTAATCCCAGCTACTTG | ACGTTGGATGTCTTGGCTCATTGCAACCTC |
| 16 | rs4788186 | 29841225 | MVP | CGCTGTGTCTATGTTCCC | ACGTTGGATGCAGCCCAGGATTACTAAAGC | ACGTTGGATGGCTAGGATTCGGCCATAAAC |
| 2 | rs4952887 | 47646968 | MSH2 | AGAAATGAGTTTCTAGGGA | ACGTTGGATGCGACTGTTTAAATAAAGTACA | ACGTTGGATGACCTACTTGCAACTATGAGC |
| 2 | rs495714 | 169779764 | ABCB11 | GCAAAAATGAAAGACAGTTCTCTG | ACGTTGGATGGACATCAGGGAGTAAGTGAC | ACGTTGGATGTCTGTGTACACCGAGGGTTC |
| 17 | rs5030740 | 1800600 | RPA1 | GATTGTGGACTAAGCAATTTCC | ACGTTGGATGTACCGATGGGTTCTGAGATG | ACGTTGGATGGTTAGCTACACAGTGCAGAG |
| 1 | rs5745532 | 76362465 | MSH4 | ACAATCTATTAGAGGAACTTTGTA | ACGTTGGATGTTCTTGCAACAGATAGGGTC | ACGTTGGATGGCCAAACCAATCTATTAGAGG |
| 5 | rs6151627 | 79965536 | MSH3 | TGGGACAAATCTGGCTC | ACGTTGGATGGCATGCTTTGCTCAAAGTGG | ACGTTGGATGGGTGTGTAATGCACAAAAGG |
| 5 | rs6151670 | 79983215 | MSH3 | TCAGTTTCCTTGAGAACTA | ACGTTGGATGGAAATGGTTGAGAGGAAAGC | ACGTTGGATGCACCTGAAAAGTCAGTTTCC |
| 5 | rs6151892 | 80118881 | MSH3 | GTTTTATGTGGAAAGATACGCT | ACGTTGGATGGAAATGAATGCCAGGCTTTG | ACGTTGGATGTGGTGGGTAGTGTTATGTGG |
| 5 | rs6151914 | 80150881 | MSH3 | CCTTCAATACATGTCTCCTTTTTTT | ACGTTGGATGGATGGAAATGACAGGGATAG | ACGTTGGATGTTCCTTCAATACATGTCTCC |
| 2 | rs6544991 | 47712780 | MSH2 | AAACAATACCACCTATTCTTAAAC | ACGTTGGATGATGATACAAAAGGATACAG | ACGTTGGATGAGTTGCCATCCCAAAGACTG |
| 2 | rs6713506 | 48038331 | FBXO11 | TGTTGCAAGATGTAAGATCA | ACGTTGGATGCCTCATTTCCCTCTGCATAC | ACGTTGGATGGAGTGAGACTCTGTCTCAAA |
| 2 | rs6742522 | 48038944 | FBXO11 | AGCCTAGGCAATGTAG | ACGTTGGATGTCAATGACCCAGTCCTGTTG | ACGTTGGATGTGAGTCTGGAGTTGGAAACC |
| 4 | rs6832850 | 174249593 | HMGB2 | CTGCTTCTCAACAATAGAAGT | ACGTTGGATGATGGAGTGTCCACTCCATAG | ACGTTGGATGTGGAGCCTGCTAAACAAAAC |
| 2 | rs6941 | 217070652 | XRCC5 | GGGTACTGTAGTAAATTTCTCAGTTTTT | ACGTTGGATGCTCTGCCATATCAGTGAACC | ACGTTGGATGGCAAGTAGAGAACAGTGAAG |
| 6 | rs707937 | 31731014 | MSH5 | ATCTGCCCTTTATATTTCCATT | ACGTTGGATGACTGAGGACCTGGAGGGCAA | ACGTTGGATGCTCTCTCAATAGATCTGCCC |
| 6 | rs707938 | 31729359 | SAPCD1 | TCTTAGCCTTGTTCAGCTACA | ACGTTGGATGTCCTCACCAAATACTGCACC | ACGTTGGATGCTTTGTGGCCACCAACTTTC |
| 16 | rs7204252 | 29864169 | MVP | CCCCAAGCTTCCCTTGTCCT | ACGTTGGATGTCCACTTCCATTCCATTGGC | ACGTTGGATGCAACTAATCTGTGTCCCAGC |
| 19 | rs7251786 | 11465129 | LPPR2 | GGTTTAAGTCATAATTTGGTGTCACA | ACGTTGGATGAAACCATGACCCCCAATTCC | ACGTTGGATGTTGGGTAAAATGCAAGGGTC |
| 12 | rs7305534 | 50341610 | AQP2 | TACGATGACACGGCTC | ACGTTGGATGCCTGGCATGAAGGGAAAGTC | ACGTTGGATGTTCTGATCTGGCCCCTAGTC |
| 12 | rs7314734 | 50340041 | AQP2 | TCTCATCATAAGAGCTCTTC | ACGTTGGATGTTAGGTATGCGCAGGCAATG | ACGTTGGATGGGTGTATCTCGTTTCTCATC |
| 7 | rs7459185 | 75934640 | HSPB1 | TCCAACCTCTCACCAGGCCCA | ACGTTGGATGTCCCCCATCTGCTTTCTACC | ACGTTGGATGCTCTCTCTTGTCTGCCCTTG |
| 3 | rs749072 | 37096024 | LRRFIP2 | CCTGCTAATCTGTAAGTATGAGAACA | ACGTTGGATGCTGGCTACCAAAAAGTGGTG | ACGTTGGATGCAAGCCGGCTAATCTGTAAG |
| 1 | rs7539638 | 210025751 | DIEXF | GTGTTTCGAATTTTTTTGGATT | ACGTTGGATGAGGAATGCCCCACCAATAAG | ACGTTGGATGTGAAATGCTTGGGACCAGAC |
| 2 | rs7602094 | 47673515 | MSH2 | CCTCTTCTAACCTCCTCCTGC | ACGTTGGATGTTCAGTAGAAGCCACCATGC | ACGTTGGATGGCACACCTATTCTAACCTCC |
| 4 | rs7686909 | 174257693 | HMGB2 | GTGTAGGGAAGTAGGAAAAC | ACGTTGGATGTTGACGTTCTCCAATCCACC | ACGTTGGATGCACCTAAAGACTGTGTAGGG |
| 5 | rs7709909 | 80001170 | MSH3 | GGCCGGAGTTTTTGGCCTGTGCTAGAGC | ACGTTGGATGCTGGTACAAAGTCCTGGAAC | ACGTTGGATGATGCCTGAGTTTTTGGCCTG |
| 8 | rs7760 | 95938422 | TP53INP1 | GATTGCAGCTTGTTTTCTTGAGCCAC | ACGTTGGATGGTCTGTGTACAAAACTGACC | ACGTTGGATGAATGGCAGCTTGTTTTCTTG |
| 13 | rs7999812 | 52545495 | ATP7B | CTTAGCAAATAAAAATATAGGACA | ACGTTGGATGTGAGCTGGACATGTTTATAC | ACGTTGGATGGGGTATCAGACTTAGCAAAT |
| 15 | rs8023369 | 58482194 | AQP9 | CAAGGGAAGAGCTTGT | ACGTTGGATGGTGGGAGTGTGAAAACATGG | ACGTTGGATGTGTGCAACTGAGAAGGATTG |
| 6 | rs805304 | 31698088 | CLIC1 | CCCAAGCCCATTCCGCCCTGCTAAGCC | ACGTTGGATGGGGCGCAGTCTGGATGTAA | ACGTTGGATGATTGTCTAAGCGGGACGGG |
| 7 | rs836548 | 6439448 | RAC1 | GAATCATACCTTTATACTTGATTTTTTC | ACGTTGGATGAGGCTTCCTTTACGCCTAAC | ACGTTGGATGCTGTTTGTACTCTAAACCAG |
| 7 | rs836554 | 6445235 | RAC1 | CTTTCACAATTGAATTCAGTACATA | ACGTTGGATGGGGATAATTAGTACTTTGC | ACGTTGGATGCAGAGCAAGACCCTTCCTTA |
| 7 | rs836556 | 6447041 | DAGLB | CAAGACGGAAAGAGGCCA | ACGTTGGATGCTTCCACATCCTAGGGTTTC | ACGTTGGATGAGTCACGAGTTTCCAAGACG |
| 1 | rs841844 | 43387302 | SLC2A1 | GGGAACTGGATCCCAGTGC | ACGTTGGATGAACCACCTTCCTCTTGAACC | ACGTTGGATGGTCTCCTCATAAAGGACTGG |
| 13 | rs873601 | 103528337 | ERCC5 | AAATACAAAGACCGTGC | ACGTTGGATGATGAATTTGTCGCAAAGACG | ACGTTGGATGTGTTTTTAGGAACCACACAC |
| 19 | rs896412 | 11444131 | RAB3D | GGTATATACAGTAGGGAACAT | ACGTTGGATGGGCATTGACAACACATTGGC | ACGTTGGATGGTTCAAAGTGCAGGAAAGGG |
| 17 | rs9082 | 1801263 | RPA1 | TAGCTTTAGTCAAAAAAAACAAAAT | ACGTTGGATGCTACCTGTACACATTCCTGC | ACGTTGGATGCAAGGGAAACAAAAGGCTTC |
| 3 | rs9852378 | 37038771 | MLH1 | TAGTAGATGAGGTCTGAGTGAAAACA | ACGTTGGATGACCCTCTAACAGTATCCCAC | ACGTTGGATGTAGGAGATGAGGTCTGAGTG |
| 3 | rs9878943 | 49434654 | RHOA | GGAGAAGCCAGTCACAAAA | ACGTTGGATGTGTGTCTCTATAGATTTGCC | ACGTTGGATGGCATAAATGAAAGAAGCCAG |
| 17 | rs9914073 | 1801592 | RPA1 | GGGAGTAAACATCAATAAAATCCCAAG | ACGTTGGATGCATTTTGCCTGAGGATGCTG | ACGTTGGATGAAGCGTGGTTGGTGTTAGAC |
| 15 | rs9920375 | 58483073 | AQP9 | ACCCTCAGATTTGTACC | ACGTTGGATGGCATTTTCCTTTTTGCATC | ACGTTGGATGTCATTGTCTTGCACCCTCAG |
| 11 | rs189037 | 108093833 | ATM | ACTACTCTCGCCTCCTCCCG | ACGTTGGATGGCTAACGGAGAAAAGAAGCC | ACGTTGGATGGTCAAAGTAGTATCAACCGC |
| 11 | rs228589 | 108093208 | ATM | AGCCTAGCCGGGTCCAATAACCCTCC | ACGTTGGATGCTTGTATTGGGTAAGCGCGG | ACGTTGGATGTTTGGCCTCAAAGGTCCTTC |
| 10 | rs2228528 | 50732280 | ERCC6 | GTCTTCAGCTCATAGTCAGTA | ACGTTGGATGAGGAAGATGACGAGGTGGAG | ACGTTGGATGGCAGAGGCTTCAGCTCATAG |
| 4 | rs2231142 | 89052323 | ABCG2 | TTTAGAAGAGCTGCTGAGAACT | ACGTTGGATGTGATGTTGTGATGGGCACTC | ACGTTGGATGCGTCATAGTTGTTGCAAGCC |
| 6 | rs462779 | 111695887 | REV3L | GGTACATCGAGAAAGCATA | ACGTTGGATGACTTAACCTCAGCACCAGAC | ACGTTGGATGAATGAGAAAGGTACATCGAG |
| 17 | rs799917 | 41244936 | BRCA1 | CCTTTGCATTTCCTGGATTTGAAAAC | ACGTTGGATGAAGGTTTCAAAGCGCCAGTC | ACGTTGGATGAGAGTGGGCAGAGAATGTTG |
| 5 | rs3776332 | 142441514 | ARHGAP26 | CCTCTGTCACATCTCATTGGTC | ACGTTGGATGCCCACAAGTGGCTCAGATAA | ACGTTGGATGTCCAGCACACTTTATGTCAC |
| 3 | rs2228000 | 14199887 | XPC | TACGGCTTGAAGAGCTTGAGGATGCC | ACGTTGGATGAAAGGCTGGGTCCAAGAGTG | ACGTTGGATGTACTGCTTGAAGAGCTTGAG |
| 7 | rs1062372 | 6048973 | PMS2 | AGGCGCGTTGGCCTTTGGCACGCGCTAC | ACGTTGGATGAGGGCTTTACCTGGTACATC | ACGTTGGATGTTTCTGAGCGTTGGCCTTTG |
| 9 | rs4647554 | 97862701 | FANCC | AGTTTTGCTGGATTAGAAG | ACGTTGGATGGGATCTATTAGCATTGCCAC | ACGTTGGATGCTCCGTTTTGAGGTATGAGC |
| 7 | rs1617640 | 100317298 | EPO | TCTCCTGGAAACCCTGAGCCAGA | ACGTTGGATGTCTAAGGTGTGAGAGACCAG | ACGTTGGATGTATGGCTTCTGGAAACCCTG |
| 6 | rs4880 | 160113872 | SOD2 | GCCCAGATACCCCAAA | ACGTTGGATGCTGTGCTTTCTCGTCTTCAG | ACGTTGGATGTTCTGCCTGGAGCCCAGATA |
| 5 | rs26279 | 80168937 | MSH3 | GGAGCAGGGACTTGTTCTGCTG | ACGTTGGATGCAGGCACAGTTTTGATCTCC | ACGTTGGATGGACAAAATCAGGGACTTGTT |
| 14 | rs175080 | 75513828 | MLH3 | TTGTTTAGAACAACAGATGC | ACGTTGGATGCAGGGTCATAGGACTTTCTC | ACGTTGGATGCAGAGAAGTTTCCATTCTCC |
| 6 | rs465646 | 111620758 | REV3L | CACTTTTTTACTTTTCAAACGAG | ACGTTGGATGGTATAAAAATCATTGCACAT | ACGTTGGATGCGAGATATCCATTCACTTT |
| 11 | rs564250 | 34458861 | CAT | AAAAACATGCTTTTTTTTATATAATGAA | ACGTTGGATGAAGAGACCTGGAGTCATTTC | ACGTTGGATGCCATAGTAACAAAAACAAAGG |
| 4 | rs615961 | 98737204 | STPG2 | CCCTGAGCACCACACACTG | ACGTTGGATGTCTCCTAATGCTATCCCTCC | ACGTTGGATGTGAGAACACATGGACACAGG |
| 11 | rs1001179 | 34460231 | CAT | TGCTCCCCGCCCTGGGTTCGGCTAT | ACGTTGGATGAGCAATTGGAGAGCCTCGC | ACGTTGGATGCTGAAGGATGCTGATAACCG |
| 2 | rs1042821 | 48010488 | MSH6 | CCGCCGCTGCCCCCG | ACGTTGGATGGCATCCCCGCCTGGGGAAG | ACGTTGGATGGCTGAGTGATGCCAACAAGG |
| 3 | rs1052133 | 9798773 | OGG1 | ACCCGTGCCGACCTGCGCCAAT | ACGTTGGATGCCCTTTGGAACCCTTTCTGC | ACGTTGGATGAGGTGCTGTTCAGTGCCGAC |
| 19 | rs1052555 | 45855524 | ERCC2 | CCTTCCACCTGGACACCCTC | ACGTTGGATGTCACAGATGCCAACCTCAAC | ACGTTGGATGTCTGCCGCAGGAAGTACTTG |
| 7 | rs1799983 | 150696111 | NOS3 | ATGCAGGCCCCAGATGA | ACGTTGGATGGGGCAGAAGGAAGAGTTC | ACGTTGGATGTGCATTCAGCACGGCTGGAC |
| 16 | rs1800566 | 69745145 | NQO1 | GGCGGTGGCTTCCAAGTCTTAGAA | ACGTTGGATGGATTTGAATTCGGGCGTCTG | ACGTTGGATGGCATTTCTGTGGCTTCCAAG |
| 3 | rs1800734 | 37034946 | MLH1 | GTGCTCACGTTCTTCCTT | ACGTTGGATGATCAATAGCTGCCGCTGAAG | ACGTTGGATGAAGTGCCTTCAGCCAATCAC |
| 13 | rs1801243 | 52548140 | ATP7B | TATGAGTTCTTCTGGGCTAATTACAG | ACGTTGGATGGTGCAGCAAATATCGGTGTC | ACGTTGGATGAGCAGCTCTGAGTTCTTCTG |
| 10 | rs2228526 | 50678717 | ERCC6 | TGCTAGTTACATTACTACTCA | ACGTTGGATGCTAATCGAAGTGATCCTTTG | ACGTTGGATGGTCTCTTCTCCAAGCCTATC |
| 2 | rs2303425 | 47630213 | MSH2 | AAGCCCTGGAAGCTGA | ACGTTGGATGTAAGCTGTTTCCCGCCTCC | ACGTTGGATGACCGAAACGCAGCCCTGGA |
| 10 | rs3740066 | 101604207 | ABCC2 | TCCACTACCTTCTCCATGCTACC | ACGTTGGATGGTCCTCAGAGGGATCACTTG | ACGTTGGATGTGTTTGATCACAAGGCCTCC |
| 11 | rs3740615 | 22647366 | FANCF | CGGAAGTAGGGCCTT | ACGTTGGATGTGCAGAAGGGATTCCATGAG | ACGTTGGATGCAATCAGTACGCAGAGAGTC |
| 17 | rs4485435 | 80045086 | FASN | GGGTGAGATGTGGACGCCTCC | ACGTTGGATGACGTGGTGGTGAGCAGGTG | ACGTTGGATGTCAGTGTGGAGCCCGGAGAT |
| 10 | rs6413432 | 135348544 | CYP2E1 | ACCCAGCTGATTAAAAATT | ACGTTGGATGCAAAGTGCCAGGATTACAGG | ACGTTGGATGCCACCACACCCAGCTGATTA |
| 2 | rs6706649 | 178130071 | NFE2L2 | AGTCACCCTGAACGC | ACGTTGGATGTTTGCCTTTGACGACCTGAG | ACGTTGGATGAGCTCGTGTTCGCAGTCACC |
| 6 | rs6941583 | 43582091 | POLH | AGGTACCGGTATGGGAT | ACGTTGGATGGGATTTCTGCAACTCCAATG | ACGTTGGATGAGTGCCCTGTGAGAAGTGTG |
| 16 | rs11646374 | 89857935 | FANCA | CGCCTGCCTGGGCCATCAAACGC | ACGTTGGATGCTCACGTCATGTTCCTGCTG | ACGTTGGATGTCTCGAATGCCTGGGCCAT |
| 7 | rs17420802 | 6017340 | PMS2 | TGAGTTCCTTGCCAACTAGTAAAA | ACGTTGGATGATCAGTTCATCGACGTCCTG | ACGTTGGATGTCACTGAAAGGGCTAAACTG |
| 1 | rs735943 | 242030151 | EXO1 | GATCGCCTGCCCATTCAAGAAGTC | ACGTTGGATGTGCTGCTAACATTAGCTGAC | ACGTTGGATGCTTTTCTAGCCTGCCCATTC |
| 14 | rs861539 | 104165753 | XRCC3 | CTCAGCTCACGCAGC | ACGTTGGATGGCTGTGAATTTGACAGCCAG | ACGTTGGATGTGGAAGGCACTGCTCAGCTC |
| 19 | rs1048290 | 10600442 | KEAP1 | GCTGTCCTCAATCGTCT | ACGTTGGATGGAATTAAGGCGGTTTGTCCC | ACGTTGGATGATGCTGACACGAAGGATCGG |
| 13 | rs1412125 | 31041595 | HMGB1 | ATTTCTTTTTAAAAGAAAATACACTAT | ACGTTGGATGTTCACCAAGACTATAATCCC | ACGTTGGATGCTACTTGAGAGTAAGCTTAG |
| 16 | rs1799801 | 14041958 | ERCC4 | CGCCATTACAGCAGATTC | ACGTTGGATGTTATACTTCTCTGACTCGGG | ACGTTGGATGGAGCTGAAACAAAGCAAGCC |
| 8 | rs1805794 | 90990479 | NBN | GGGGTGAATTCCTGAAAGCAGTT | ACGTTGGATGCTTTCAATTTGTGGAGGCTG | ACGTTGGATGGGACGTCCAATTGTAAAGCC |
| 10 | rs2228527 | 50678369 | ERCC6 | TCAAACTTGGCGTCTC | ACGTTGGATGCTGAGACCAAAGCAAAAGCC | ACGTTGGATGTTCACCAGGTGTGGAATTCG |
| 3 | rs2229032 | 142178144 | ATR | GTGTCTGAAAAACTCAAAGTATTCC | ACGTTGGATGGGAATGTTCTCAGAAACCAC | ACGTTGGATGCCAAAGTCAGCAGCTTTATC |
| 17 | rs2243828 | 56358884 | MPO | CTCCTCTCACCATTGTGTGCCTATACC | ACGTTGGATGGGTTAGTTGTGTGTATCCCC | ACGTTGGATGCACTACCAGCCCAAGATTTC |
| 13 | rs2249825 | 31037903 | HMGB1 | ACCAGTACTTTGGTTTTCATTCC | ACGTTGGATGGACATATAAGACCTTAAAGT | ACGTTGGATGAGGTTGATGTCGCTACTGTG |
| 10 | rs2273697 | 101563815 | ABCC2 | GTCAGGTTCACTGTTTCTCCAA | ACGTTGGATGTATCCAACTTGGCCAGGAAG | ACGTTGGATGTGGTCACATCCATGAGCTTC |
| 17 | rs2333227 | 56358762 | MPO | CCGACCTCAAGTGATCCACC | ACGTTGGATGAGTGGCTCATGCCTGTAATC | ACGTTGGATGTGAACTCCTGACCTCAAGTG |
| 8 | rs2735383 | 90947269 | NBN | GTTTGATCACCAGAGTTTAGGTAAGA | ACGTTGGATGTTTAATATTTTTTCTTTTCC | ACGTTGGATGGCACCACTGAAGCCATTTTG |
| 2 | rs3087386 | 100055506 | REV1 | TCTCCGGCTTTCTCCAGCCT | ACGTTGGATGCTCTGAAATCAGTGCTGCTC | ACGTTGGATGATGGTCAACAGTGTTGCCAG |
| 7 | rs3213619 | 87230193 | ABCB1 | AAGCCTGAGCTCATTCGAG | ACGTTGGATGCTAAAGGAAACGAACAGCGG | ACGTTGGATGTTTGCCACAGGAAGCCTGAG |
| 10 | rs3793784 | 50747539 | ERCC6 | AGAAGCAGGACAGCTCT | ACGTTGGATGTTAGCCGCACTTTTTGGTCG | ACGTTGGATGGGGAACAGAGAAGCAGGAC |
| 6 | rs3823434 | 35426175 | FANCE | GACTTGCCCTTTCACCTGATCTCAGCCT | ACGTTGGATGAATACACCTACCCGTCCAAG | ACGTTGGATGCTGGCCCTTTCACCTGATCT |
| 17 | rs4246445 | 80039028 | FASN | GCAGCGGTGCGTGTGGG | ACGTTGGATGACAACAAGGAGGAAGGGCTG | ACGTTGGATGTGCAGGAGCTGTCCTCAAAG |
| 12 | rs5744751 | 133253995 | POLE | GGGTCAGATACCGAGGAAATG | ACGTTGGATGGGTCGTTCAACAAGGTCATC | ACGTTGGATGGTGTTTTGCAGGCTCATTGG |
| 7 | rs6464268 | 152381150 | XRCC2 | TTGGGCTTTTCTGTGTGCACATATACC | ACGTTGGATGAAGTGAAACGTGGAGGATGC | ACGTTGGATGGGCCTAACTGTGCTTTTCTG |
| 11 | rs7943316 | 34460472 | CAT | GAAGACTGAAGTCGCCACGG | ACGTTGGATGTCCACCCTCAGCAGGCAAAT | ACGTTGGATGCTGATTGGCTGAGCCTGAAG |
| 19 | rs11545829 | 10599965 | KEAP1 | GGGCAGCGTGGAGCGCTA | ACGTTGGATGTACGAAAGTCCACGTCTCTG | ACGTTGGATGACTGTATCTATGCTGCTGGG |
| 11 | rs4585 | 108239628 | ATM | CTTGTAAAAGACTATTCTAGTCTTTACG | ACGTTGGATGGCAAATAAAAGCAAAGAGG | ACGTTGGATGCAAACCAATATACTGGCTTTC |
| 3 | rs10342 | 129155670 | MBD4 | GAAGCTGTGTGTAATAAAGCAGAT | ACGTTGGATGCCAGCATCAGAAATGCAGAC | ACGTTGGATGAGCAAAAGAGAATCTGTGTG |
| 19 | rs25489 | 44056412 | XRCC1 | TGCCAGCTCCAACTC | ACGTTGGATGTGTCACTGCCCCCTGTGCT | ACGTTGGATGTTTGTCTTCTCCAGTGCCAG |
| 3 | rs140693 | 129155451 | MBD4 | CTCCGCCAAAGACTCAGAACACAAC | ACGTTGGATGCAACTTCTACTTTTGTTCCG | ACGTTGGATGTTGTTCAGCCAAAGACTCAG |
| 13 | rs543304 | 32912299 | BRCA2 | CGATTTTCTATCTTAAACATTGAAAC | ACGTTGGATGGCAGAGGTACATCCAATAAG | ACGTTGGATGCTTACAGTTTTATCATTATG |
| 11 | rs769217 | 34482908 | CAT | CCAACTACCAGCGTGA | ACGTTGGATGTTAGGCCTACCCTGATTGTC | ACGTTGGATGTACCTGTGAACTGTCCCTAC |
| 5 | rs1650697 | 79950781 | DHFR | CCCGTCCCAGACAGAACCTACTA | ACGTTGGATGTTCCCGCCCCAGCTGCCG | ACGTTGGATGTCCGCCCGCCTTACCCACAA |
| 8 | rs1801195 | 30999280 | WRN | GAATTGTGTCCAAAGAAGTT | ACGTTGGATGGCAACAAAAAATGCAGAAG | ACGTTGGATGCCTCATCCTTCAAGCTAATG |
| 15 | rs1801321 | 40987565 | RAD51 | CTTTGGTCGGGCGCGTGCCAC | ACGTTGGATGCCGCGCTCCGACTTCACCC | ACGTTGGATGGTAGAGAAGTGGAGCGTAAG |
| 10 | rs2031920 | 135339845 | CYP2E1 | GCTTAATTCATAGGTTGCAATTTT | ACGTTGGATGTCATTTCTCATCATATTTTC | ACGTTGGATGGTTCTTAATTCATAGGTTGC |
| 17 | rs2048718 | 59940819 | INTS2 | GGATAGGAAAGGTAACGGCGGCCCCA | ACGTTGGATGTTCCTCCTCCCTCTTCCTG | ACGTTGGATGTGGGTCGAGGAAAGGTAACG |
| 6 | rs2075789 | 31708328 | MSH5 | TGGGGCCCGGCACTG | ACGTTGGATGAAGGAGGACACCGCAGGGA | ACGTTGGATGACTTCCTCCTCCTCGGCCT |
| 3 | rs2227928 | 142281612 | ATR | AGAATTTATTGAAGTCACTTTATTAA | ACGTTGGATGCACAATTGCAATAATACGAG | ACGTTGGATGGCTCCTTTGCAGTTGATGAG |
| 3 | rs2228001 | 14187449 | XPC | CCCCCACCTGTTCCCATTTGAG | ACGTTGGATGAACTGGTGGGTGCCCCTCTA | ACGTTGGATGCCCAAGAAGACCAAAAGGGA |
| 1 | rs2233004 | 11742074 | MAD2L2 | TTATTGCCCTCAGAGATGATATTA | ACGTTGGATGTGACACATAAGAAGTGCTCG | ACGTTGGATGTGTAAGGGCCCTCAGAGATG |
| 16 | rs2239359 | 89849480 | FANCA | CCACCCCTGGTTCCC | ACGTTGGATGGGCCAATGAGATGTAGTCTG | ACGTTGGATGATCCTCAGGTGCACATTCTC |
| 3 | rs2272125 | 10138069 | FANCD2 | AAAAAGACCCTGGAACT | ACGTTGGATGTAGATTGCCCAGCCAGAAAG | ACGTTGGATGTGCCTCTGCTCAAAAAGACC |
| 17 | rs2297518 | 26096597 | NOS2 | ATCTTTCTAGAAACTGAAGAAAT | ACGTTGGATGAGTAGGACAACGGAAAAAGC | ACGTTGGATGCCCTCTCTTTCTAGAAACTG |
| 14 | rs2307486 | 20924204 | TMEM55B | TCCACATTCCAAGAGCAGA | ACGTTGGATGAACCTCACCCAGTGGCAAAC | ACGTTGGATGAAGCCCATCCACATTCCAAG |
| 11 | rs4442551 | 22648843 | FANCF | TCTGTAGCACCTCATACTA | ACGTTGGATGAGCAAATGTACAGGCATCAC | ACGTTGGATGCTACTAAATCTGTAGCACCTC |
| 11 | rs4447177 | 22646025 | FANCF | AGAGGAAGTGATTGGAAGTA | ACGTTGGATGACTATTGCTAATTCCATGGC | ACGTTGGATGAGTAGCTGGCATGTAGTGTC |
| 17 | rs4986765 | 59763465 | BRIP1 | ATTTGAAAGTGCACTGGA | ACGTTGGATGGGTTCTGTCCTTTATGGATAC | ACGTTGGATGTCAGCACCATTCAACCTTTG |
| 6 | rs6907678 | 35418954 | FANCE | CGGAAAGCCCAACTCCT | ACGTTGGATGCCAGGTCAGATCTTAAGAGG | ACGTTGGATGACTCCCATGGCTTTCCATAC |
| 17 | rs11079454 | 59757169 | BRIP1 | GGCCATTTTCATTACTGATAAC | ACGTTGGATGTGTGCCAGGTGCTCTTTTAA | ACGTTGGATGGTAGGCCATTTTCATTACT |
| 8 | rs13312840 | 90997909 | NBN | GCTTCGGAGTTTGCATCTATGATGA | ACGTTGGATGATAGAGCAAGACCCTGTCTC | ACGTTGGATGTGTGCTTAGGAGTTTGCATC |
| 13 | rs206118 | 32889792 | BRCA2 | AACCCGCTCCAGAGG | ACGTTGGATGCGGTTTTTGTCAGCTTACTC | ACGTTGGATGCCACTACCACCACCACTAAC |
| 3 | rs373572 | 8955389 | RAD18 | GAACAGCTGCTGAAATAGTTC | ACGTTGGATGAGACGCATCCTAGTCTTCTC | ACGTTGGATGGACATGTGATTAACCTAGTGG |
| 10 | rs717620 | 101542578 | ABCC2 | GGGACTGGACTGCGTCTGGAAC | ACGTTGGATGCCTGTTCCACTTTCTTTGATG | ACGTTGGATGAGCATGATTCCTGGACTGCG |
| 1 | rs746218 | 11743124 | MAD2L2 | GGGGTTTGCATTGGTGACAGGCA | ACGTTGGATGAGTCTGAAGGGCTCCTTTGC | ACGTTGGATGGGCTGCTTAAGCAGCTTTTG |
| 1 | rs1047840 | 242042301 | EXO1 | TGAAGAGTCCTACTCTTTT | ACGTTGGATGAAGTGGGTGGTGAAATGGTC | ACGTTGGATGCAAGGCAACAGTGTTTACAG |
| 3 | rs1050450 | 49394834 | GPX1 | CGAATCCCTGCTGTCTCAAGGGC | ACGTTGGATGACTGCAACTGCCAAGCAGCC | ACGTTGGATGTTGACATCGAGCCTGACATC |
| 12 | rs1051669 | 1022452 | WNK1 | ATCAGAATGAAGCAAGATAAAT | ACGTTGGATGTTCATCGCTGCTGGAGAATG | ACGTTGGATGCTCTCAGTCAGATCCTCTTG |
| 17 | rs1140616 | 80039481 | FASN | GGCCGTGGCACACAT | ACGTTGGATGCTCCTACTGGGAGTGCTC | ACGTTGGATGTATAGGGACAGGGACAGCCA |
| 10 | rs1649942 | 83951691 | NRG3 | AAGTGTTCATGTATTAGAATCGAA | ACGTTGGATGTTGTTTCCTAGCTGCCTCTG | ACGTTGGATGGAGAGAATGAAAGGAAAGTG |
| 1 | rs1776148 | 242042545 | EXO1 | CTCATCCCTTACGAGAAG | ACGTTGGATGACTTTCCTGGGACTGTGAAG | ACGTTGGATGCAGTTAAAGAGCGAGGAGTC |
| 9 | rs1805329 | 110084328 | RAD23B | CTCCAGCAGCTAGTACTGGGG | ACGTTGGATGTGTTGCTGTCGTAGTTGCTG | ACGTTGGATGAGAAAGTCAGGCTGTGGTTG |
| 7 | rs2070744 | 150690079 | NOS3 | CCAAGCTCTTCCCTGGC | ACGTTGGATGAGGTCAGCAGAGAGACTAGG | ACGTTGGATGACTGTAGTTTCCCTAGTCCC |
| 1 | rs2185383 | 208779683 | PLXNA2 | TAGGCTTGTGTGGGG | ACGTTGGATGGATCAGTTTTAGGTTCACAG | ACGTTGGATGTGATAATGGAGGAGGCTAGG |
| 8 | rs2230009 | 30921935 | WRN | CAACCTGACATGGAAGAAA | ACGTTGGATGCAGTTGTGTGTTTCTGAGAG | ACGTTGGATGTGTAGAGATACCAACCTGAC |
| 5 | rs2230641 | 86695274 | CCNH | GTAGGAACCACCCAGATCTGAAGAAG | ACGTTGGATGTGCAAGCTCAGCAGAATGAC | ACGTTGGATGAAGAAGTATGAACCACCCAG |
| 1 | rs2233006 | 11741796 | MAD2L2 | CGGCTGGAGCTCCAAAA | ACGTTGGATGCGCCCTACGCTTCATAACAA | ACGTTGGATGAATCCACGGACACACTTAGC |
| 2 | rs2303428 | 47703500 | MSH2 | CCGTCCCTCCCATATTGGGGCCTACA | ACGTTGGATGCCATTTATTAGTAGCAGAAAG | ACGTTGGATGGTTGATTTACCTCCCATATTG |
| 1 | rs3219489 | 45797505 | MUTYH | GGGTGCAGGCACAGGTGGCA | ACGTTGGATGGTTCCCAGCTCCCAACAC | ACGTTGGATGGGAAGTTGACCACTCCCAG |
| 6 | rs9462088 | 35430686 | FANCE | CCCTATCAATACTCACGTTAG | ACGTTGGATGTATGCCAAGCTCATGCTGAC | ACGTTGGATGCCAAGGCCCTATCAATACTC |
| 15 | rs12593359 | 41023878 | RAD51 | TCGGCACAAGACTCCATAACCAAA | ACGTTGGATGCAGTAAAACTCTCAAGCAGG | ACGTTGGATGTAGGTTTGGCACAAGACTCC |
| 11 | rs12806698 | 4115974 | RRM1 | TAATTCGCCTGTCAGTCTGTGAAG | ACGTTGGATGAATGACGTTACTCGACGCTG | ACGTTGGATGACTCAACATGGCGGCTACAC |
| 2 | rs35652124 | 178130073 | NFE2L2 | CCGTGGGAGTTCAGAGG | ACGTTGGATGAGCTCGTGTTCGCAGTCACC | ACGTTGGATGTTTGCCTTTGACGACCTGAG |
| 19 | rs238406 | 45868309 | ERCC2 | CCTGCCCCACTGCCG | ACGTTGGATGTACCTGTCCTGCCTCCCTC | ACGTTGGATGCAGTACCAGCATGACACCAG |
| 17 | rs1042522 | 7579472 | TP53 | CAGAGGCTGCTCCCC | ACGTTGGATGTAGGAGCTGCTGGTGCAGG | ACGTTGGATGCAATGGTTCACTGAAGACCC |
| 14 | rs1130409 | 20925154 | TMEM55B | TTCTGTTTCATTTCTATAGGCGA | ACGTTGGATGACAATCACCCGGCCTTCCTG | ACGTTGGATGCTTGATTGCTTTCCCTTTTC |
| 1 | rs1136410 | 226555302 | PARP1 | CAGGTTGTCAAGCATTTCC | ACGTTGGATGGCTTTCTTTTGCTCCTCCAG | ACGTTGGATGTGCTATCATCAGACCCTCCC |
| 14 | rs1799794 | 104179267 | XRCC3 | ACCAAGTTCTCAGCAGG | ACGTTGGATGATAACAGACTCACCGGTTGG | ACGTTGGATGGCCTGTTAAACCAAGTTCTC |
| 15 | rs1801320 | 40987528 | RAD51 | GTGGAGCGTAAGCCA | ACGTTGGATGCGCTCCGACTTCACCCCGC | ACGTTGGATGAAGCGAGTAGAGAAGTGGAG |
| 7 | rs2228006 | 6026775 | PMS2 | CTGAAAAAGAGTCGTCAGTTT | ACGTTGGATGAGGAACATGTGGACTCTCAG | ACGTTGGATGTGGTTTGAATGGCAGTCCAC |
| 17 | rs2228309 | 80051183 | FASN | TTGGGCTTCAGCAGGAC | ACGTTGGATGAGAACGCCTACCAGGCCAT | ACGTTGGATGAGAACTGCACGGAGGTGTTG |
| 19 | rs2298881 | 45926916 | ERCC1 | CCCCCCCCCGCCTTCCGTT | ACGTTGGATGAGAGATGGACAAGGCCAGG | ACGTTGGATGATTCTATTGGCTCCGTCCCC |
| 3 | rs3218649 | 121208833 | POLQ | GGGAATCAAGAACATCAGA | ACGTTGGATGAAAGAGGCCCGTTTTCTTGC | ACGTTGGATGCCAGAATGGGAATCAAGAAC |
| 15 | rs4417527 | 41021280 | RAD51 | CTGTGCAAATATTTTAATAAAACAT | ACGTTGGATGAAAGTGCTGGGATTACAGGC | ACGTTGGATGAACGGTGCCTCAGATGTTAG |
| 17 | rs4986764 | 59763347 | BRIP1 | ACCTCTTTAAAGTACAGTACC | ACGTTGGATGATAGATGACTTGCTGCTTCC | ACGTTGGATGGGACAATGAGTCTACACTTG |
| 2 | rs5742933 | 190649316 | PMS1 | TCACACCACACTACCTT | ACGTTGGATGCGGCTAGTGGATGGTAATTG | ACGTTGGATGATACCCGTTAGTCACACCAC |
| 2 | rs6721961 | 178130037 | NFE2L2 | ATGGGAGATGTGGACAGC | ACGTTGGATGCTTAGGAGAATGGAGACACG | ACGTTGGATGCCTGCCTAGGGGAGATGTG |
| 12 | rs7963551 | 1021515 | WNK1 | GGGCATGAAACAGAGTTTTTGTAT | ACGTTGGATGCCCTCTTCAGCTGCAGTGTA | ACGTTGGATGTGGAATTCACCTGTGTTTCG |
| 16 | rs9282681 | 89805914 | FANCA | AGCCCCGGATCAGCAC | ACGTTGGATGGGAGGTACCTGTAAAAAGCG | ACGTTGGATGTGTTGCAGACCTCAGGCTGG |
| 11 | rs11020802 | 94227125 | MRE11A | CAAGAGGCCCCGCCCTCA | ACGTTGGATGTCCTAGCCTGCTCAGGATTG | ACGTTGGATGTTCGGCTCTCATTGGCTACC |
| 4 | rs230529 | 103457418 | NFKB1 | CTTAAGAGGTAATTCCTTTGTTTTG | ACGTTGGATGCAGTGTTTGCTTACAAAAGGC | ACGTTGGATGGAAGTTTCAGAGGTAATTCC |
| 6 | rs316003 | 160645832 | SLC22A2 | AGCAACCAAGCCAAG | ACGTTGGATGGTGATATTCATCTGTTTGGC | ACGTTGGATGGTTTCTGGAAGCAACAGCAC |
| 6 | rs316019 | 160670282 | SLC22A2 | GTAAGAAGTTGGGCAGAG | ACGTTGGATGCTTACGCACTTCCTCACTGG | ACGTTGGATGGGACTTACCAGTAATAGAGC |
| 13 | rs1045411 | 31033232 | HMGB1 | TGCCAAGATAGTTACAGTGCT | ACGTTGGATGCTTCATTTTAAAAGTTGGCCC | ACGTTGGATGTTAATGCCAAGATAGTTAC |
| 13 | rs1061472 | 52524488 | ATP7B | TCCCCCAGGGACCACC | ACGTTGGATGTAAGTGGCGTTTGTTGCAGG | ACGTTGGATGATCCACTGGAAACTTTCCCC |
| 1 | rs1061622 | 12252955 | TNFRSF1B | CCAAGTGCAGACTGCATCC | ACGTTGGATGTGGTGGCCATCCCTGGGAAT | ACGTTGGATGGGTAAGTGTACTGCCCCTG |
| 4 | rs1585215 | 103444474 | NFKB1 | GTGCAGATTTGCAGGGAAAC | ACGTTGGATGGTGGCTGTCTGTATATCTTC | ACGTTGGATGAGCTCTGAGTCCTTCAGAAC |
| 6 | rs1800629 | 31543031 | TNF | CCCCGAGGCTGAACCCCGTCC | ACGTTGGATGGGTCCCCAAAAGAAATGGAG | ACGTTGGATGGATTTGTGTGTAGGACCCTG |
| X | rs2227291 | 77268502 | ATP7A | TTGCCTACTCTTTGATTATTCTTCTA | ACGTTGGATGTAGGGTTCACTTTGGCTCTC | ACGTTGGATGGGCAACCACCATTGCATTTG |
| 14 | rs2233406 | 35874799 | NFKBIA | GGGGTTGTGGATACCTTGCAATA | ACGTTGGATGGTAGGTCAGATAGCATAAACG | ACGTTGGATGGTAGTGGTGGTTGTGGATAC |
| 12 | rs2234649 | 6451363 | TNFRSF1A | AGCACAGGAGTCCCAGA | ACGTTGGATGTTGGTGTTTGGTTGGGAGTG | ACGTTGGATGTAGTCCAGTCACAAGCACAG |
| 12 | rs2279744 | 69202580 | MDM2 | GGGGCTGCGGGGCCGCT | ACGTTGGATGAGCGTTCACACTAGTGACCC | ACGTTGGATGTTCAGGGTAAAGGTCACGGG |
| 17 | rs2289669 | 19463343 | SLC47A1 | GGTCTAGCCGGGAACT | ACGTTGGATGCCAGTTTGTGCTAAGCATCG | ACGTTGGATGAACATCCCCTTTGTCTAGCC |
| 12 | rs4149570 | 6451590 | #N/A | ACTTCTCAGACACATAACTGAA | ACGTTGGATGACAGGTTATCTCCACTCTGC | ACGTTGGATGAGCTAAGAATGTGTCTTGGA |
| 4 | rs4648068 | 103518305 | NFKB1 | GAAGCTAATTGTTAGAGATTCCA | ACGTTGGATGCACCAATATCTTGGTGAAA | ACGTTGGATGGTTTATGGCAGAGATGTGTG |
| X | rs6622665 | 77277268 | ATP7A | ACCTGCATCTGTCAATA | ACGTTGGATGGTACCTAAGATACAGGATTGG | ACGTTGGATGTTGGCTGTCACCTGCATCTG |
| 13 | rs9535826 | 52566126 | ATP7B | TTTTAAAAGGCATAACTGGTAA | ACGTTGGATGGAAATGTGCCCTCCAGACTC | ACGTTGGATGTGTTTCAGACAGGCAGAGAG |
| 13 | rs9535828 | 52573422 | ATP7B | AGTCCTTCCCTCCCC | ACGTTGGATGAGAACAGTAGCAAAGAGCAG | ACGTTGGATGTTAAAGCCCACCAGTCCTTC |
| 14 | rs11549465 | 62207557 | HIF1A | CGCGGAACTGCTTTCTAATG | ACGTTGGATGCTTCCAGTTACGTTCCTTCG | ACGTTGGATGCTTTGAGGACTTGCGCTTTC |
| 12 | rs11614913 | 54385599 | HOXC10 | ACTCGGCAACAAGAAACTG | ACGTTGGATGTCGACGAAAACCGACTGATG | ACGTTGGATGCTGATCTGTGGCTTAGGTAG |
| 17 | rs28392491 | 18373249 | LGALS9C | GGGAAATGCAGGTGCTCGGCCTGA | ACGTTGGATGCAGAACTTTGGGTTGACCAC | ACGTTGGATGTAAAAGAATGCAGGTGCTCG |
| 6 | rs28688207 | 32628660 | HLA_DQB1 | TGGCTCTGCATAACTTCCTTTTGTA | ACGTTGGATGAATATTACCTGCTGGTGGAG | ACGTTGGATGTGAGAGAGTGGCTGTTTGTG |
| 7 | rs2280497 | 45747933 | ADCY1 | AGCCTAGGTGTAAACAGA | ACGTTGGATGCTTGCACTCCCCAGATGTAA | ACGTTGGATGGGAGGAGATGGACTTCTTAG |
| 7 | rs2293106 | 45753324 | ADCY1 | AAAGTGGTAGGGGCA | ACGTTGGATGTCAACAGGTGACTGAGGAAG | ACGTTGGATGTTGACACTGACTTTGCCTCG |
| 2 | rs2290158 | 175287778 | SCRN3 | GGAGCCTGATCCTGAGAGGT | ACGTTGGATGGACCCCTTGCTGGTTTATAG | ACGTTGGATGGCCTTCCTTGCATTCACTTC |
| 19 | rs2306190 | 14162676 | PALM3 | CTGGCAAGACAGGAG | ACGTTGGATGACATTCATGAACCCTGCACC | ACGTTGGATGTTTGTGCCTTAGGTGGTAGC |
| 5 | rs2549794 | 96244549 | ERAP2 | GGCTAAGATTTAAACTGAATGG | ACGTTGGATGGAGAACAATAACAGAAGTAGG | ACGTTGGATGTTACTCAGCTAGTCAGTGGC |
| 11 | rs2659870 | 6653232 | DCHS1 | CCCTAACCAGGCTGGGGATAAGGCTATG | ACGTTGGATGTGAGCACAGAGACCCAAATC | ACGTTGGATGCTGTCTGAATGTTCACCAGG |
| 5 | rs3733977 | 167957554 | FBLL1 | CCCCAGAAAATAATAGCAAACACAA | ACGTTGGATGAGAGCAGCAGCAAATAGCAC | ACGTTGGATGCTCACAAAGAAAACACACAG |
| 15 | rs3809581 | 32620127 | LOC102724099 | CCTCAAGGAAAAGATACACATTCTTC | ACGTTGGATGGAACCAAGTTTTGAGCCTAC | ACGTTGGATGGAGTTAGAGAATTCTCTGCC |
| 4 | rs6816526 | 164449273 | 42430 | GGTTGTTGCGTGACTTAAACAAAC | ACGTTGGATGTGAAGACATTGATATTTGG | ACGTTGGATGCTGTGGGTATTTAACAGAGC |
| 2 | rs10496029 | 54399058 | ACYP2 | CCACCCAACCCAAGAATATC | ACGTTGGATGAAGGTGCCGTTTTCTTTCCC | ACGTTGGATGAGGATTACAGGTGTGAACCG |
| 3 | rs12487651 | 46714708 | ALS2CL | AGTGTCTCCTCCCAA | ACGTTGGATGTTTGCCTCATGATTGCTGGG | ACGTTGGATGGTCAGAATGACAGTGTCTCC |
| 2 | rs10175949 | 234712921 | MROH2A | GGGGTCTAATCACATGACTTGAATAAC | ACGTTGGATGCTGTGTGGGTCAACTGTGAG | ACGTTGGATGTCCAATCCGTGTATGTGCTG |
| 6 | rs1042544 | 33054457 | HLA_DPB1 | GTCTTTGAATCAAAGAGCAGAA | ACGTTGGATGGCCAAGAAGTTGCTCTGAAG | ACGTTGGATGAGAAGGGAACATGGTTGGAG |
| 6 | rs117780937 | 32629685 | HLA_DQB1 | AAGAGATGGGAAGGAATGG | ACGTTGGATGTTACTATGGGCCCACAAGAC | ACGTTGGATGCAGCTCAGTAGTGACATCAG |
| 6 | rs117988605 | 32628705 | HLA_DQB1 | AAAGGTCTCATTACACAAACAG | ACGTTGGATGGGGTGGGAAGAGAATGTAAC | ACGTTGGATGGCAGAGAAAGGTCTCATTAC |
| 16 | rs11859599 | 82182832 | MPHOSPH6 | TTGAAAGCTGCCGTG | ACGTTGGATGTAGCATTCATTGGCTCTCCC | ACGTTGGATGGATCACAGTCATCGTCCTAC |
| 3 | rs12488699 | 33866868 | PDCD6IP | AGGGGTCTTTTTGTTGCATGTGAAAA | ACGTTGGATGCAGCTCGACATTGAACATAT | ACGTTGGATGGAGGTAACTCCAATTTATC |
| 6 | rs149415995 | 32487205 | HLA_DRB5 | ATCCACTTGGCAGGTGTAAAC | ACGTTGGATGGGTGATGCTGGAAACAGTTC | ACGTTGGATGACGCTTGGGTGCTCCACTTG |
| 11 | rs2278900 | 9495961 | ZNF143 | GGGGAGCAGGGAAGCTTTACTGAACTT | ACGTTGGATGCTATACTTGCTTCATGAGTC | ACGTTGGATGCCTTTAAACACTGACGCAGG |
| 6 | rs28366217 | 32557328 | HLA_DRB1 | CTGCCATGTCCCTTAAGAAA | ACGTTGGATGAAGATGGGCAATCTCTGAAG | ACGTTGGATGTTGTTAACATTGTGCCCAGG |
| 6 | rs3204373 | 32632801 | HLA_DQB1 | TGTGCTACTTCACCAA | ACGTTGGATGTATCTGGTCACAAGACGCAC | ACGTTGGATGCAGAGGATTTCGTGTTCCAG |
| 6 | rs3213487 | 32724317 | HLA_DQB2 | GGAGATGTTTGATGCTACAGAGG | ACGTTGGATGACGAGGCATGATCAGCACAG | ACGTTGGATGACCTCAGCATCCTCTGTTTG |
| 6 | rs34236112 | 32634394 | HLA_DQB1 | CCCCACTGCTTTTCCCTT | ACGTTGGATGGCAAAGCCTTCTTCCAAGAC | ACGTTGGATGACATCAGATCCATCAGGTCC |
| 6 | rs34309628 | 32549331 | HLA_DRB1 | TGCACAGTGGAATGGAGT | ACGTTGGATGTGACACTCAGGGATTAGCAC | ACGTTGGATGCAAGCCCTCTCACAGTGGAA |
| 6 | rs9272422 | 32605132 | HLA_DQA1 | TGTGGCCAAAACCTGAC | ACGTTGGATGGAAGACACCCAAACCAAACC | ACGTTGGATGCTAGTAACTGAGATGTCACC |
| 6 | rs9272742 | 32609722 | HLA_DQA1 | GCAGAGCTATTCACACTT | ACGTTGGATGAACCTCTGTGGTGAGGAAAC | ACGTTGGATGGCTCTTCCAGGATGTAATGC |
| 6 | rs9273528 | 32628633 | HLA_DQB1 | GGGGACTGGATCATGGCTGAAATAT | ACGTTGGATGTGTAAGACCTCAAGGGCCTC | ACGTTGGATGTTCCCTCTTATACCTGTGCC |
| 7 | rs2163938 | 48415892 | ABCA13 | ATATCACTGGCCATAACT | ACGTTGGATGTAGTTAACAATCCCCCCACC | ACGTTGGATGAGAAGGTCAATATCACTGGC |
| 19 | rs2227270 | 18420704 | LSM4 | CGAGCAGGGGAGCAGGTTATTAACTTAC | ACGTTGGATGTGCAGGCTGGGCAGGATCT | ACGTTGGATGGAGAAGGGGAGCAGGTTATT |
| 3 | rs2276713 | 119205541 | POGLUT1 | GACCCAGTATACCCCTC | ACGTTGGATGCAATGACTCTGCACAAGTGG | ACGTTGGATGACCTAGATGCTGCAGTACCC |
| 17 | rs2302293 | 66987202 | ABCA9 | TTCCAATTTTTGGAGTAGATTCATTC | ACGTTGGATGTGTTTGAAAGGCGGATGGTG | ACGTTGGATGCTTCCAATTTTTGGAGTAG |
| 19 | rs2903755 | 33098870 | ANKRD27 | GGCCACATACTTCTAAACAAATT | ACGTTGGATGACAGCTCCCACACACAATGC | ACGTTGGATGTTTGGCATGCCACATACTTC |
| 17 | rs3213690 | 66246416 | AMZ2 | GTTGTTCCCCAGCAT | ACGTTGGATGACCCAGTGCTTGTATCACAG | ACGTTGGATGTCCAAAGAGATCACTGGCTG |
| 6 | rs6915736 | 152762098 | SYNE1 | TGTTTTACTTCTTGATGTTTAATC | ACGTTGGATGCCCATTCTTTCAACATATTGG | ACGTTGGATGGGGAACTAACCATTTTCCAA |
| 6 | rs9483504 | 133135886 | RPS12 | GGTTTTTTTTTAGTGCGTTCAAGATTCA | ACGTTGGATGTCGGCCATGGCGGTGGGTTA | ACGTTGGATGTCGTGACGAGTATCTGGTTC |
| 9 | rs10970975 | 32431931 | ACO1 | GGGCCCTCAAGGCTAACGG | ACGTTGGATGACCTCCCCAGTCATCTGAAG | ACGTTGGATGAAAGCTGCTCCCTTTGTGTC |
| 1 | rs11205255 | 153234602 | LOR | ACACGACAAATACTCTTGGAGACCTAAA | ACGTTGGATGCCCAGTGCCTCAGTCAATAA | ACGTTGGATGTGGATGAACTTGAACACTAC |
| 5 | rs12654410 | 167957055 | FBLL1 | CTTCCCTGGGCGCCGCGTC | ACGTTGGATGAATGATGTCGGAGACATGGG | ACGTTGGATGATCCACATCAAGCCCAAGTC |
| 5 | rs13163821 | 80655709 | ACOT12 | GATTTGATGGTCAGTAAATCC | ACGTTGGATGAAGACTCCTTTGCCTGTCAC | ACGTTGGATGGTAGCAAATTTGATGGTCAG |
| 8 | rs28485205 | 82370696 | FABP9 | GGAGGCAAAGAGGAATCTATTAGAG | ACGTTGGATGCAGGGCAAGTTTAGCCTGTT | ACGTTGGATGTCGTAGATTCTGGTGCTGAC |
| 2 | rs60558096 | 54398837 | ACYP2 | ACTTACTCTATTATTAAAATTTCCAGTT | ACGTTGGATGGAGCTTTTTTCACCCCTTTC | ACGTTGGATGCAACTTCCAAAGAAGCTTAG |
| 7 | rs61128227 | 120773954 | CPED1 | TTGAAATAAGTGGGAATATCTATA | ACGTTGGATGGTGCCATTTGATGCAATAGA | ACGTTGGATGGTTTTGCATTTTTTTGCCAGG |
| 7 | rs143565372 | 45776474 | SEPT7P2 | AGGACTTGAAAGATGTTACTAATAAT | ACGTTGGATGAGTCACAGCTGCCAGTTTTC | ACGTTGGATGACACACGCAGGACTTGAAAG |
| 6 | rs1136633 | 32487170 | HLA_DRB5 | GAGCACCCAAGCGTGA | ACGTTGGATGGAAAGCTGCTCACTCCATTC | ACGTTGGATGAGTGGAGAGGTTTACACCTG |
| 6 | rs144532965 | 32489795 | HLA_DRB5 | CTCGGTTCCAGTACTCAGCG | ACGTTGGATGAAGAGGAGGACTTGCGCTTC | ACGTTGGATGTCCTTCTGGCTGTTCCAGTA |
| 6 | rs28366215 | 32557310 | HLA_DRB1 | ACAATGATAAAGATGGGCAATC | ACGTTGGATGCAGGCCATGTCCCTTAAGAA | ACGTTGGATGGTACCCAAGCTCCTTTTATG |
| 6 | rs28366218 | 32557374 | HLA_DRB1 | CCTGGGCTACTATGGGGTGGGGAAAA | ACGTTGGATGTTCTTAAGGGACATGGCCTG | ACGTTGGATGACGTAAGTGCACATTGCGGG |
| 9 | rs991121 | 123370345 | MEGF9 | TTTTAAGGACTAGTAGTTGTTTT | ACGTTGGATGTTAGTACAGTCTGAAGCTAC | ACGTTGGATGTTGGCCTCCAAAGTTCTGGG |
| 3 | rs2633851 | 4403817 | SUMF1 | GTGGACTGGGGAAGACT | ACGTTGGATGGCCCACTATGGACTGACAAC | ACGTTGGATGAGAAAAGCCCAATGTAGGTC |
| 16 | rs3859104 | 55895249 | CES5A | TTCTATGTTCCAAATTCAAATAAATA | ACGTTGGATGCTTCGAACGGAGAGATGAAC | ACGTTGGATGCAAGTACCTCAATCCTCCTC |
| 10 | rs1244229 | 8007560 | TAF3 | TTGAGGAGAAAGAGAAGG | ACGTTGGATGTCTTCTTCTCCTTTTTGTCC | ACGTTGGATGTCTTTGTTGCCAGTGCTTCC |
| 9 | rs12985 | 32450187 | ACO1 | GGGAGTGGTGGAGAGGCCTCCC | ACGTTGGATGTTGCTCCATCTACAAGGCAG | ACGTTGGATGAAGCCGCACCACCAGCCAG |
| 3 | rs3792594 | 33877626 | PDCD6IP | CAATCAATCGTGCCCTT | ACGTTGGATGGAAGTCATTATCCTTCTTTG | ACGTTGGATGAACAGTGGCATCTCGCTATG |
| 16 | rs9937572 | 55890398 | CES5A | CCTCGGTAAGCCATCTGGTCAT | ACGTTGGATGCAACTTCCCCTGTGAAAGTC | ACGTTGGATGAGAAGCGTGCCCTCTGTAAG |
| 3 | rs1060407 | 47958037 | MAP4 | AGCAGAGGATATTTCTGTG | ACGTTGGATGAGAAATAGAGGTGGCACAGG | ACGTTGGATGTTTCTGAGGACAAAGCCACC |
| 6 | rs11545686 | 32605288 | HLA_DQA1 | CCCCCGCTCTGACCACCGTGA | ACGTTGGATGCTCACCCACAATGTCTTCAC | ACGTTGGATGAAACAAAGCTCTGCTGCTGG |
| 11 | rs12417980 | 89531540 | TRIM49 | GAATCAGAATGAGAAGATAGAT | ACGTTGGATGACCCAAGAAGAAAGAGTCCC | ACGTTGGATGGGAATTGGGCTTTTGGTGTC |
| 4 | rs13130399 | 164466824 | 42430 | CCACATTCCACGTAATCGC | ACGTTGGATGGCTGTCCGGTCTATCAATAC | ACGTTGGATGTCTGCTCTGTCACATTCCAC |
| 4 | rs1561736 | 164440581 | TMA16 | AGCCGTGCAAGAGGAAAACTA | ACGTTGGATGCGTTTAGTTCCAATTCCCCC | ACGTTGGATGGATGCAATTCCCAAGACGTG |
| 2 | rs2234500 | 71160222 | VAX2 | AAGGCAGATCCAGGAGC | ACGTTGGATGTCTGCCAGCTGTCTGCTTTT | ACGTTGGATGTCTAGCCAGCTGTATGGCTC |
| 16 | rs2303262 | 82203758 | MPHOSPH6 | CAGTAGATTCTTGGACAAC | ACGTTGGATGGCTGGGCGGAAGCTACCAT | ACGTTGGATGCCTTCATGCGCAGTAGATTC |
| 4 | rs2916467 | 4249415 | TMEM128 | ATACGAGTCGACCTGA | ACGTTGGATGAAATATCTGCCTTGCCCGTC | ACGTTGGATGCCCATTGGTACATACGAGTC |
| 14 | rs34652473 | 106471595 | IGHV1-3 | GCCCCCTTGACTGGC | ACGTTGGATGAAATCCCAGTGAGGAGGAAG | ACGTTGGATGTGGACACCTGTAGAGAAGAC |
| 2 | rs36004074 | 186665432 | FSIP2 | CCCTCCCTTACGATAATCAGATTTT | ACGTTGGATGAGAGGAAACATCAGCAGAAG | ACGTTGGATGACAGTCCATTCCTCCCTTAC |
| 7 | rs4236392 | 5487458 | FBXL18 | GACGCTGAGGAGCCCCC | ACGTTGGATGACAAGCTGCCTCTTCAACCC | ACGTTGGATGTGAGCTTCTCGCCAGGTCTC |
| 9 | rs4836833 | 123632829 | PHF19 | CGGCTGAAAGAGAGG | ACGTTGGATGACTTCCATTCCTGTGAGCTG | ACGTTGGATGTAGGCAGATGTTGCACTTGG |
| 1 | rs61801822 | 161565324 | FCGR2C | CCCCTAGTGCCCTGGCTAATC | ACGTTGGATGATCAGTGGAATTGGCTGTGG | ACGTTGGATGTTTTGCAGCCTCAGCATCAG |
| 12 | rs63281060 | 19609952 | AEBP2 | CTAAAACATGCTGGAGCCAAGTT | ACGTTGGATGTTCCACTGGTATTGCCATCC | ACGTTGGATGATGGTGACAACATGCTGGAG |
| 11 | rs649870 | 118971251 | DPAGT1 | GGAAGTCTCCTCCAAGTGTTCCTTTT | ACGTTGGATGACGATAGGATGAAGGGCTAC | ACGTTGGATGTCCACATTCTCCTCCAAGTG |
| 1 | rs67170285 | 241838057 | WDR64 | CAGTTTTTAATAACCAGGTAATTT | ACGTTGGATGTGTAAAATGAGTCATTCAG | ACGTTGGATGATAACAGCTACTCAGAAAGG |
| 15 | rs7496668 | 100821576 | ADAMTS17 | AAGCAGCCGACGTGGGGCAGGCCTT | ACGTTGGATGTGGTGAGCCGGATAGCGTTC | ACGTTGGATGGAAAAGAAGAAGCCGACGTG |
| 12 | rs7964052 | 68715417 | MDM1 | CTCGCGTCTATTTTATTACTCTGAAATG | ACGTTGGATGAATGTGTAACATCTTAAGC | ACGTTGGATGTTAATTCCATCTCTGCTTCC |
| 6 | rs7990 | 32609965 | HLA_DQA1 | GGAGACTTGCAGTCATAAATCTCATCA | ACGTTGGATGTCAGTTACCTCACCTTCCTC | ACGTTGGATGAGTGTTTCAGAAGAGGCTGG |
| 7 | rs12666778 | 77261820 | PTPN12 | GAAAAACATACCTGATTTTAATCTA | ACGTTGGATGGCATGTCAACATCAATTTAC | ACGTTGGATGAACTCCGAAAAACATACCTG |
| 8 | rs12674488 | 6338306 | MCPH1 | AGGGAACCAGACGTCTGTGAGA | ACGTTGGATGATTCCCAGCAGCACATTCAG | ACGTTGGATGCTTTTCAATTGCACCAGACG |
| 22 | rs2073774 | 19028480 | DGCR2 | GAAATCCGGAAAGAAGTGAGGTCCC | ACGTTGGATGTTGTCAAGTAACCTGGCACG | ACGTTGGATGGACAAAGCCGGAAAGAAGTG |
| 6 | rs2294757 | 133035098 | VNN1 | GGGACGCTGCCAGGACACTTTCA | ACGTTGGATGGGTGGCATTGGGCAATATCG | ACGTTGGATGTCTATGTCTCAAGAGCCAGC |
| 2 | rs3732268 | 70046262 | ANXA4 | GTATGTGAAAATACATAGAGCATC | ACGTTGGATGCCAGGGAAATTTTAGCCAAG | ACGTTGGATGGAGGGTACTTTCTATCTGTG |
| 2 | rs62128457 | 26467347 | HADHB | GCCGGGGCCACCCCAGT | ACGTTGGATGAGACCTCCGGGCAGAACTC | ACGTTGGATGAGGCGGCAGCGAAAGGGAGA |
| 17 | rs736523 | 73588067 | MYO15B | CCCTCTGTACAGGTGGAGGAT | ACGTTGGATGTGGCATTGAGGATGGTCTTG | ACGTTGGATGCTTGAACACCCCTCCCTGTA |
| 6 | rs7385 | 76425594 | SENP6 | TACCTAAAATTGCAACTTCTAAAC | ACGTTGGATGGAGGAATTAAACAAGTTCAC | ACGTTGGATGTAGTACCTAAAATTGCAAC |
| 6 | rs7740756 | 96655105 | FUT9 | TGTGTGTGTGTGTGTA | ACGTTGGATGTGAGTTAACTAGATCCAGAG | ACGTTGGATGCTATTGTCACTAGCTGTGGG |
| 6 | rs77866376 | 32487175 | HLA_DRB5 | CCAAGTGGAGCACCCAAG | ACGTTGGATGAGTCAGAAAGCTGCTCACTC | ACGTTGGATGAGAGGTTTACACCTGCCAAG |
| 8 | rs2929970 | 134241137 | WISP1 | GGAAGATGGAGGTTTACC | ACGTTGGATGGCTTCAACCTCTTCAGCTTT | ACGTTGGATGTTCTGGTAGGAAGATGGAGG |
| 8 | rs2977530 | 134215112 | WISP1 | TCCTCTGAGTCAGCCA | ACGTTGGATGAGGTGTTGGGAAAAGAGGTG | ACGTTGGATGTCCCCACGCTTGTTTCAAAG |
| 8 | rs2977549 | 134242033 | WISP1 | TTTAACAGTCAAAATTTTATATTAAGTG | ACGTTGGATGCATACATATGCATTTCTTTG | ACGTTGGATGCCAAAGCTACATGAAAATAG |
| 8 | rs116716037 | 134203336 | WISP1 | AGAGGCCCAGAGGATCCGAC | ACGTTGGATGATATCTGGTGCTCCTGATGG | ACGTTGGATGTCAGTGGCACAGGCATCGAC |
| 8 | rs2929973 | 134242508 | WISP1 | GCAGGGAAATTCTGACTTAGAAAG | ACGTTGGATGCCATGGAGAAGGAAATTTGG | ACGTTGGATGTGAATCAGGCCACACCATTG |
| 8 | rs2013146 | 134202992 | WISP1 | CATCCTGCACCCTCCCA | ACGTTGGATGTCATCCTGCCTAGGTCTTTC | ACGTTGGATGGTAACAGTATCTCCTCGGAC |
| 8 | rs2929946 | 134220691 | WISP1 | TCCGACACAAATCTAACCCTGCC | ACGTTGGATGGCTGAGGTCTGGAAGAATGG | ACGTTGGATGGGACTGGATACAGTGTGGTG |
| 8 | rs2977529 | 134214906 | WISP1 | CCCTACCGCACTGCCCAGAA | ACGTTGGATGCAACTTTCTGGAAACCACCC | ACGTTGGATGGTGTATGAGCCAGGTACATC |
| 8 | rs4330674 | 134221502 | WISP1 | GCCTGCAGACCCAAA | ACGTTGGATGATGTCCAGCATAGACGCTTA | ACGTTGGATGAGAGAGGTGAGGATTGATCG |
| 8 | rs2977536 | 134219277 | WISP1 | ATTTACTCTAAGCCTGTGCTTACA | ACGTTGGATGGGGAAACTATGGCTTCACTC | ACGTTGGATGAGGACTCTAGTCTGAAGTGC |
| 8 | rs16904853 | 134232303 | WISP1 | AAAATTGTAACTGCCAACATTCCA | ACGTTGGATGCAGACAAAAGACATGCCCTC | ACGTTGGATGTCCCAGACACTTGTAACTGC |
| 8 | rs2929965 | 134235816 | WISP1 | TCTCTTCTTTTCTGTGTTTATCTTTT | ACGTTGGATGAAGAATGAAGGGAGGGAAAG | ACGTTGGATGCCTCTTTATCCTTCTAATCC |
| 8 | rs2977519 | 134208839 | WISP1 | GAAAAGGTAGGGAGGGAGAA | ACGTTGGATGCAAAGCCTGTAAGTGGAGAC | ACGTTGGATGCAAGTGTGCCTAAGGCTATG |
| 8 | rs10956697 | 134230782 | WISP1 | TGTCCTGTCTCTGTTATTGGATACTG | ACGTTGGATGGTAATGCAATGCACAATACAG | ACGTTGGATGATGTCTAGCAGGCATGAAGG |
| 8 | rs3739261 | 134239770 | WISP1 | GGATTACTGTGGAGTTTGCATGGACAA | ACGTTGGATGGGACACGTCGATAGTCTTAG | ACGTTGGATGTCAACCCAAGTACTGTGGAG |
| 8 | rs3739262 | 134205713 | WISP1 | AAGCATAAGTCATATTTGCAATACA | ACGTTGGATGGCCGAGTGTATTTGGGAATT | ACGTTGGATGCATGCTCAACAAATGCAGAAG |
| 8 | rs16893344 | 134206279 | WISP1 | CACACCAAGCTCCCTG | ACGTTGGATGTCCTCAGGCACATTTTCACG | ACGTTGGATGGACCTCACTTTCCTCGTTTG |
| 8 | rs10956696 | 134226879 | WISP1 | GGGATGACCCCCTGCTTCTGTC | ACGTTGGATGGCTCTCTTAATCTGATGAGTC | ACGTTGGATGTGAAACTTTCTGACCCCCTG |
| 8 | rs112897791 | 134197982 | WISP1 | AGAGGAGCTGGGACTACAGGTGCC | ACGTTGGATGAACCCCGTCTCTACAAATAC | ACGTTGGATGCTCTGGAGTAGCTGGGACTA |
| 8 | rs11778573 | 134228930 | WISP1 | CTCATTCCACCAAACCACACTG | ACGTTGGATGTTAAATGAAGCTGGGTCAGG | ACGTTGGATGGCAGAGCCAGGATCTAAAAC |
| 8 | rs138013484 | 134240098 | WISP1 | CGGTCTCTAACCATTCAAATGA | ACGTTGGATGAAACTCATAGCATGGGCCTG | ACGTTGGATGTTGGCCTCCATTTCTGTCTC |
| 8 | rs142461254 | 134240594 | WISP1 | GGTGGGGACAAACCTAATCTGTA | ACGTTGGATGTGGGCATTGTTGAGGTTAAG | ACGTTGGATGATTTCTGACTGGGACAAACC |
| 8 | rs146162433 | 134241416 | WISP1 | TGACTCTGGTTGAGTTTTC | ACGTTGGATGCAAATGTGAAACGGAAGCTC | ACGTTGGATGTCCTAACTTTCCCAGTCACC |
| 8 | rs16904845 | 134199776 | WISP1 | ATTATTTCCCCAAGTAGAGCC | ACGTTGGATGCTACTGAATTCATTCATTCC | ACGTTGGATGGATCTGTGCAGCTTCAAACA |
| 8 | rs2013158 | 134202942 | WISP1 | GGGACCCCAGGCAGGCGG | ACGTTGGATGACAGGGAGTTGATTTCCCAC | ACGTTGGATGGTCCGAGGAGATACTGTTAC |
| 8 | rs2929969 | 134240697 | WISP1 | GAGCGTCAATTGGTCCTTCTGAAA | ACGTTGGATGAAGCCCCCAGTTAATACTCC | ACGTTGGATGGCTGATTCAGTGTGAGAGTC |
| 8 | rs2929986 | 134211526 | WISP1 | AGTTAAAAAGGAAACAAGAAGAATG | ACGTTGGATGTACCCTGCCTAGAGCACAAG | ACGTTGGATGCATCTCTGAGCCTCAATGTC |
| 8 | rs2977537 | 134220063 | WISP1 | GTGAATGTTCATTTCCCCAAC | ACGTTGGATGGTTTCTGCCTTCAAAGGATG | ACGTTGGATGAAGCATCTGTTGATAGGGTG |
| 8 | rs2977551 | 134243550 | WISP1 | TGGGAATCATAAGTCAACTATGTATC | ACGTTGGATGCACAGTTTGAAAATAGATAC | ACGTTGGATGACCCTGCATATCTGGGAATC |
| 8 | rs35472615 | 134197537 | WISP1 | GTACCCTTGCACTGGAGGTGGGGC | ACGTTGGATGTCAGAGCTGGATGCAGGGAT | ACGTTGGATGGCCTGCATTTAACCTTGCAC |
| 8 | rs4265166 | 134241086 | WISP1 | CCCTTCCATTTGGTCAGCAGAAC | ACGTTGGATGGTTCAGGACACATCTATTGC | ACGTTGGATGCTTCCTACCAGAAAACTGGC |
| 8 | rs62514003 | 134198196 | WISP1 | GGGTTTGAGCCCTGGGAGAAGTCAC | ACGTTGGATGAGACAAGGGAACCAGAACAC | ACGTTGGATGGAACTTCTACCTCCTGCTAC |
| 8 | rs62514004 | 134202489 | WISP1 | CCCATTTCATTTGCCCTCTTGCAC | ACGTTGGATGAAAGCCACCTCTTTCGCTTG | ACGTTGGATGATTACAGGCGTGAGGCATTG |
| 8 | rs72731505 | 134198960 | WISP1 | CAGAGGGTTCATGGC | ACGTTGGATGTCAGAGAGGGACCCTGTGTA | ACGTTGGATGGTGATTTATTGGTACCGGTG |
| 8 | rs72731507 | 134200038 | WISP1 | CCCTAGACACTTAGTGTTTGCCC | ACGTTGGATGGCTACGATTGGGTTGTTGTG | ACGTTGGATGTATTGTTCTGGTCCACTGGC |
| 8 | rs754958 | 134238771 | WISP1 | TGAATGCTTACTGTATACCAAG | ACGTTGGATGCCCTGTAATACTAGCTTTTG | ACGTTGGATGTGGTACCTTTTCCATGTACG |
| 8 | rs7828685 | 134224979 | WISP1 | GGTTTTCATAAAATTATTTTTTCTATCA | ACGTTGGATGAAAGGAGATGTTTGGCTATC | ACGTTGGATGTTCCTGTCCTCATGCTGTTC |
| 8 | rs7843546 | 134198301 | WISP1 | CGGGCTTGCCACCTTG | ACGTTGGATGTGAACCATCAGCCACATGAG | ACGTTGGATGGGCCTGTTAAGGAATTCCTC |
| 19 | rs11615 | 45923653 | ERCC1 | GAAGTTCGTGCGCAA | ACGTTGGATGATAGTCGGGAATTACGTCGC | ACGTTGGATGTTGATGGCTTCTGCCCTTCG |
| 11 | rs1695 | 67352689 | GSTP1 | AGGCCCTCCGCTGCAAATAC | ACGTTGGATGGCAGATGCTCACATAGTTGG | ACGTTGGATGTGGTGGACATGGTGAATGAC |
| 7 | rs1045642 | 87138645 | ABCB1 | GCTTTGCTGCCCTCAC | ACGTTGGATGGCTGAGAACATTGCCTATGG | ACGTTGGATGTATGTTGGCCTCCTTTGCTG |
| 13 | rs1047768 | 103504517 | ERCC5 | AGTCCGGGATCGCCA | ACGTTGGATGGCAGAGCCGATGAAACAAAG | ACGTTGGATGCAAGCACTTAAAGGAGTCCG |
| 19 | rs13181 | 45854919 | ERCC2 | GGGAGTCAGAGGAGACGCTG | ACGTTGGATGCACCAGGAACCGTTTATGGC | ACGTTGGATGAGCAGCTAGAATCAGAGGAG |
| 13 | rs17655 | 103528002 | ERCC5 | TAAAGATGAACTTTCAGCAT | ACGTTGGATGACCTGCCTCTCAGAATCATC | ACGTTGGATGTTCGCAGCTGTTCTCCTTTG |
| 19 | rs1799793 | 45867259 | ERCC2 | CCCTGCAGCACTTCGT | ACGTTGGATGACGGACGCCCACCTGGCCAA | ACGTTGGATGAGGCGGGAAAGGGACTGGG |
| 9 | rs1800975 | 100459578 | XPA | GGAGGCCGCCGCCATCTC | ACGTTGGATGGTGGAGCTGGGAGCTAGGT | ACGTTGGATGTCCGCGGGTTGCTCTAAAG |
| 7 | rs2032582 | 87160618 | ABCB1 | AGATAAGAAAGAACTAGAAGGT | ACGTTGGATGCATATTTAGTTTGACTCACC | ACGTTGGATGTGTTGTCTGGACAAGCACTG |
| 19 | rs25487 | 44055726 | XRCC1 | CCGGCGGCTGCCCTCCC | ACGTTGGATGAGGATAAGGAGCAGGGTTGG | ACGTTGGATGTAAGGAGTGGGTGCTGGACT |
| 19 | rs3212986 | 45912736 | ERCC1 | ATGGACAAGAAGCGGAAG | ACGTTGGATGCTTTAGTTCCTCAGTTTCCC | ACGTTGGATGCACAGGCCGGGACAAGAAG |
| 11 | rs227091 | 108237839 | ATM | TCTCCTGCCTCAGCCTCC | ACGTTGGATGAAAATTAGCCGGGCGTGTTG | ACGTTGGATGCTGGGTTCAAGCAATTCTCC |
